# Blockage of glutamine-dependent anaplerosis affects mTORC1/2 activity and ultimately leads to cellular senescence-like response

**DOI:** 10.1242/bio.038257

**Published:** 2019-05-16

**Authors:** Geng-You Liao, Ming-Ting Lee, Jhen-Jia Fan, Pei-Wen Hsiao, Chun-Sheng Lee, Shou-Yi Su, Jiuan-Jiuan Hwang, Ferng-Chun Ke

**Affiliations:** 1 Institute of Molecular and Cellular Biology, College of Life Science, National Taiwan University, Taipei 106, Taiwan; 2 Institute of Biological Chemistry, Academia Sinica, Taipei 115, Taiwan; 3 Agricultural Biotechnology Research Center, Academia Sinica, Taipei 115, Taiwan; 4 Institute of Physiology, School of Medicine, National Yang-Ming University, Taipei 112, Taiwan

**Keywords:** Amino-oxyacetate, Cellular senescence, Glutamine-dependent anaplerosis, Metabolism, mTORC, p16^INK4A^

## Abstract

The purpose of study was to explore the role of glutamine-dependent anaplerosis in cell fate determination (proliferation and senescence) and the potential associated mechanism by employing a pharmacological inhibitor of glutamine-dependent anaplerosis, amino-oxyacetate (AOA). Using the WI38 normal human embryonic fibroblast cell line, we found that exposure to AOA induced mTORC1 inactivation−mTORC2 activation (within day 1), cell cycle arrest (day 2–6) and cellular senescence (day 4–6). These AOA effects were blocked by concomitantly providing anaplerotic factors [α-ketoglutarate (αKG), pyruvate or oxaloacetate], and not affected by ROS scavenger N-acetyl-cysteine (NAC). Moreover, AOA-induced cellular senescence in WI38 cells is associated with elevated protein levels of p53, p21^CIP1^ and p16^INK4A^ and decreased Rb protein level, which was blocked by αKG supplementation. In p16^INK4A^-deficient U2OS human osteosarcoma cells and p16^INK4A^-knockdown WI38 cells, AOA exposure also induced similar effects on cell proliferation, and protein level of P-Rb-S807/811 and Rb. Interestingly, no AOA induction of cellular senescence was observed in U2OS cells, yet was still seen in p16^INK4A^-knockdown WI38 cells accompanied by the presence of p16 antibody-reactive p12. In summary, we disclose that glutamine-dependent anaplerosis is essential to cell growth and closely associated with mTORC1 activation and mTORC2 inactivation, and impedes cellular senescence particularly associated with p16^INK4A^.

## Abstract (precis)

**Summary:** Glutamine-dependent anaplerosis is essential to cell growth and closely associated with mTORC1 activation and mTORC2 inactivation, and impedes cellular senescence particularly associated with p16^INK4A^.

## INTRODUCTION

Cellular metabolic reprogramming is one of the critical challenges during cell growth and proliferation. Proliferating cells require specific metabolic activities to convert nutrients into energy and biosynthetic building blocks that are essential to replicate all of the macromolecular components needed for the construction of new cells. In both normal and tumor cells, those undergoing proliferation rewire their central metabolic pathways, especially aerobic glycolysis (also termed the Warburg effect) and mitochondrial cataplerosis-and-anaplerosis, to shunt metabolites into biosynthetic pathways for biomass accumulation, including lipids, heme and amino acids (Bauer et al., 2005; Hatzivassiliou et al., 2005; Pizer et al., 1996). Mitochondrial cataplerotic activity relies greatly on glutamine-dependent anaplerosis through incorporating glutamine-derived αKG to replenish the pools of the tricarboxylic acid (TCA) cycle and maintain mitochondrial functions (DeBerardinis et al., 2008; Owen et al., 2002). Although glutamine-dependent anaplerosis is essential to sustain mitochondrial functions and support cell growth; little is known about the potential contribution of glutamine-dependent anaplerosis in coordinating nutrient-sensing pathway and cell growth.

Mechanistic target of rapamycin (mTOR) acts as a key signaling hub that couples growth-related pathways with growth factor signaling and nutrients availability (Guertin and Sabatini, 2007). Currently, mTOR is known to form two main structurally and functionally distinct kinase complexes: mTORC1, which controls biomass accumulation by regulating translation and autophagy (Bar-Peled and Sabatini, 2014; Cohen and Hall, 2009), and mTORC2, which controls cytoskeleton organization by regulating the actin cytoskeleton (Hagiwara et al., 2012; Jacinto et al., 2004; Kumar et al., 2010; Sarbassov et al., 2004). mTORC1 is mainly regulated by growth factors and nutrient availability. Growth factors mainly activate mTORC1 through the well-characterized PI3K-AKT-TSC1/2-Rheb signaling pathway (Laplante and Sabatini, 2012). On the other hand, several mechanisms have been proposed for the regulation of mTORC1 by nutrient availability (Bar-Peled and Sabatini, 2014; Cohen and Hall, 2009; Fingar and Blenis, 2004; Zoncu et al., 2011). For example, upon amino acid refeeding in starved cells, mTORC1 is activated by Rag GTPases and Ragulator (Sancak and Sabatini, 2009). In addition to nutrient availability, metabolic activity is equally important for biomass accumulation and cell growth. Growing evidence reveals that metabolic pathways play important roles in regulating mTORC1 activation. For example, the metabolism of glutamine and glucose regulate mTORC1 activity by controlling its complex assembly through the TTT-RUVBL1/2 complex (Kim et al., 2013). These findings suggest the importance of nutrient availability and metabolism in regulating mTORC1 activity to ensure proper coordination to meet the demand of energy and building blocks for cell growth. mTORC2 is also regulated by growth factors and nutrients (Guertin and Sabatini, 2007) while the associated mechanism is poorly identified.

In mammalian cells, proliferation is strictly restricted by various stresses such as DNA damage to ensure systematic homeostasis (de Magalhães and Passos, 2018). Cells facing continuing growth-limiting conditions may undergo cell cycle arrest, cellular senescence or apoptosis (Childs et al., 2014). Cellular senescence, the ultimate and irreversible loss of replicative capacity in normal cells, may serve as a tumor suppressor and a contributor to aging and certain age-related diseases (Campisi et al., 2011). In the 1960s, Hayflick and Moorhead observed that normal human fibroblasts lost their ability to proliferate in cell culture and referred to this phenomenon as replicative senescence (Hayflick, 1965; Hayflick and Moorhead, 1961). Unlike reversible quiescence, cellular senescence is characterized by specific phenotypes that includes irreversible growth arrest, flat cell morphology, senescence*-*associated β*-*galactosidase activity (SA-β-gal)*,* formation of senescence-associated heterochromatic foci (SAHF) and upregulation of the p53/p21^CIP1^ and/or p16^INK4A^ pathways (Dimri, 2005; Narita et al., 2003). For cellular senescence and organismal aging, mitochondrial dysfunction has been implicated as the critical factor (Beckman and Ames, 1998; Chen et al., 1995; Shigenaga et al., 1994; Sohal and Weindruch, 1996; Wallace, 1999). Of note, it has been reported that growth factor signals are required to trigger the cellular senescence response (Takahashi et al., 2006). Upon growth factor, the reprogrammed mitochondrial metabolism is not only required to produce energy but also to provide biosynthetic precursors for cell growth (DeBerardinis et al., 2008; Lunt and Vander Heiden, 2011). Emerging evidence implicates that the impaired metabolic pathway, which leads to the imbalance of mitochondrial metabolites, may play roles in triggering senescence (Borradaile and Pickering, 2009; Hashizume et al., 2015; Ho et al., 2009; Jiang et al., 2013; Kaplon et al., 2013; Langley et al., 2002; Lee et al., 2012; van der Veer et al., 2007). In proliferating cells, glutamine-dependent anaplerosis is a critical pathway of the mitochondrial metabolism and is essential for cell growth and cell cycle progression, yet little is known regarding the role of a sustained impairment of glutamine-dependent anaplerosis in the induction of cellular senescence.

Here, we used amino-oxyacetate (AOA), a pan-aminotransferase inhibitor frequently used to suppress glutamine-dependent anaplerosis (Kaadige et al., 2009; Wise et al., 2008; Wise and Thompson, 2010), alone or in combination with anaplerotic factors αKG, pyruvate or oxaloacetate (DeBerardinis et al., 2008; Owen et al., 2002), to evaluate the role of glutamine-dependent anaplerosis in mTORC signaling and cell fate determination (cell proliferation and cellular senescence). On the basis of the importance of glutamine-dependent anaplerosis in the macromolecular biosynthesis required for cell growth and mTORC1's central role in coordinating the anabolic processes and nutrient availability, we were intrigued to understand whether glutamine-dependent anaplerosis plays a critical link of glutamine availability and metabolism to mTORC1 activity and cell fate determination.

## RESULTS

### Inhibition of glutamine-dependent anaplerosis with AOA leading to cell cycle arrest, mTORC1 inactivation and mTORC2 activation is not mediated by ATP depletion in WI38 normal human embryonic fibroblast cell line

**Fig. 1.**
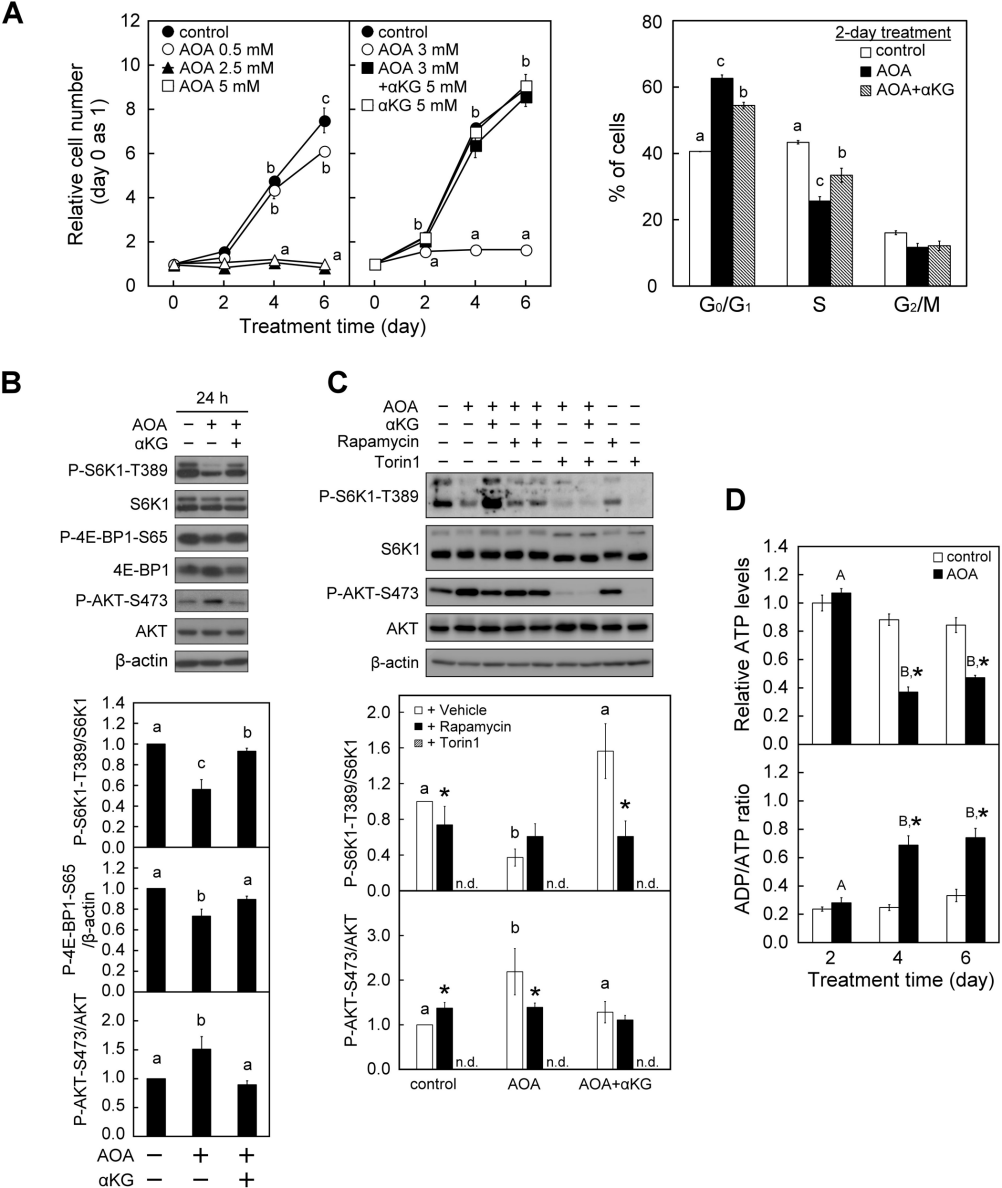
**Inhibition of glutamine-dependent anaplerosis with AOA induces inhibition of cell proliferation and cell cycle arrest in WI38 cells.** WI38 cells were treated with vehicle or AOA in the absence or presence of 5 mM αKG for the indicated time-period with medium changed at 2-day intervals. (A) Effect of AOA on cell proliferation and cell cycle progression. Left panel: the relative cell numbers were calculated by normalizing against the value of day 0. Right panel: cell cycle analysis was conducted after a 2-day treatment period using flow cytometry with Propidium Iodide staining of DNA. The percentages of cells in various phases of the cell cycle are presented. (B) Effect of AOA on mTORC signaling. Cell lysates were prepared after a 1-day treatment period and analyzed for the activation status of mTORC1 and mTORC2 by immunoblotting and densitometry analysis of P-S6K1-T389/S6K1 and P-4E-BP1-S65/β-actin, and P-AKT-S473/AKT with β-actin served as a loading control. (C) Effect of mTOR inhibitors on AOA-regulated mTORC1/2 activities. Cells were first treated with 3 mM AOA and then given vehicle (DMSO), 1 nM rapamycin or 30 nM Torin1 1 h before the end of the 24-h culture period. Cell lysates were analyzed by immunoblotting as described in B. (D) Effects of AOA on energy status. Intracellular ATP content and ADP/ATP ratio were determined by ApoGlow Assay Kit. All quantitative data are expressed as the mean±s.e.m. (*n*=3) of three independent experiments. Different lowercase letters indicate significant difference among treatment groups at the same time-point (*P*<0.05). Asterisk (*) designates a significant difference compared with the respective vehicle control (*P*<0.05).

To investigate the role of glutamine-dependent anaplerosis on cell growth and proliferation, WI38 cells were chronically exposed to AOA to suppress glutamine-dependent anaplerosis by inhibiting the conversion of glutamate to αKG (Hensley et al., 2013; Kaadige et al., 2009; Wise et al., 2008; Wise and Thompson, 2010). Treatment of WI38 cells with AOA dose-dependently suppressed the proliferation of these cells with near complete suppression at 2.5 to 5 mM observed after 2 days and throughout the 6-day culture period (Fig. 1A, left panel). Accordingly, 3 mM AOA was used for the following experiments. To further examine whether the AOA effect involves perturbation of glutamine-dependent anaplerosis, cells were supplemented with αKG. αKG is the cellular intermediate of glutamine supply to the TCA cycle, and αKG could enter cells through secondary active transporters of the SLC13 family−Na^+^-dependent high affinity dicarboxylate transporters (NaDCs) (Kekuda et al., 1999; Liu et al., 2010; Pajor, 2014). Importantly, simultaneous supplementation with 5 mM αKG remarkably prevented the AOA-induced inhibition of WI38 cell proliferation (Fig. 1A, middle panel), which confirms the specificity of the anaplerosis-blocking activity of AOA. In addition, cell cycle analysis reveals that treatment of WI38 cells with 3 mM AOA for 2 days resulted in an increase of cells accumulated in the G_0_/G_1_ phase compared with the vehicle control, and this was mitigated by concomitant supplementation with αKG (Fig. 1A, right panel). Together, these results show that glutamine-dependent anaplerosis is indispensable for G_1_ cell cycle progression and therefore proliferation in WI38 cells.

Given that coordinating regulation of both mTORC1 and mTORC2 is critical for cell growth (Laplante and Sabatini, 2012; Oh and Jacinto, 2011), we investigated whether there was a correlation between mTORC activity and glutamine-dependent anaplerosis. Treatment of WI38 cells with AOA significantly reduced the mTORC1 activity as indicated by the decreased phosphorylation of S6K1 at Thr389 (P-S6K1-T389/S6K1) and 4E-BP1 at Ser65 (P-4E-BP1-S65) (Fig. 1B). S6K1 and 4E-BP1 are downstream targets of mTORC1 that are frequently used as indicators of mTORC1 activity (Sarbassov et al., 2005a). At the same time, mTORC2 was activated following AOA treatment as indicated by the increased phosphorylation of AKT at Ser473 (P-AKT-S473/AKT) (Fig. 1B), a widely used indicator of mTORC2 activity (Jacinto et al., 2006; Sarbassov et al., 2005b; Shiota et al., 2006). Moreover, concomitant supplementation with αKG significantly attenuated AOA-induced mTORC1 inactivation and mTORC2 activation compared with the treatment with AOA alone (Fig. 1B). To further consolidate whether AOA disturbance of glutamine-dependent anaplerosis is closely associated with mTORC1 inactivation and mTORC2 activation, two types of mTOR inhibitors (rapamycin and Torin1) were used. Rapamycin is an allosteric mTOR inhibitor with greater sensitivity (lower doses and shorter treatment time-period) toward mTORC1 than mTORC2 (Li et al., 2014; Sarbassov et al., 2006). Torin1, on the other hand, is an adenosine tri-phosphate (ATP)-competitive mTOR inhibitor which similarly blocks mTORC1 and mTORC2 activity (Thoreen et al., 2009). WI38 fibroblasts were treated with vehicle control, AOA and/or αKG (AOA±αKG) for 24 h, and rapamycin or Torin1 was given 1 h before the end of culture. We have confirmed the specificity and dose-dependent effect of rapamycin and Torin1 on mTORC1 activity (P-S6K1-T389/S6K1) and mTORC2 activity (P-AKT-S473/AKT) in WI38 fibroblasts (Fig. S2). Rapamycin significantly reduced mTORC1 activity in the control and AOA+αKG-treated cells, increased mTORC2 activity in the control, and decreased the AOA-induced increase of mTORC2 activity (Fig. 1C). Interestingly, we noticed that cells given rapamycin appear to have similar mTORC1 and mTORC2 activity regardless of the treatment (control, AOA or AOA+αKG). On the other hand, Torin1 markedly suppressed both the mTORC1 and mTORC2 activity in control and AOA±αKG-treated WI38 cells (Fig. 1C). Together, the above results support the concept that AOA disruption of glutamine-dependent anaplerosis is closely associated with mTORC1 inactivation and mTORC2 activation in WI38 normal human embryonic fibroblasts.

Glutamine-dependent anaplerosis plays a critical role in fueling mitochondrial respiration, which contributes to ATP production (Fan et al., 2013), and mTORC1 activity is sensitive to energy status (Gwinn et al., 2008; Inoki et al., 2003; Kim et al., 2013). We therefore examined the effect of AOA on energy status of the cells. WI38 cells were exposed to 3 mM AOA for the indicated time-periods, and the cellular ATP contents were determined using a luciferase assay (Goto et al., 2006). Although AOA treatment reduced the mTORC1 activity within 24 h (Fig. 1B), there were no significant changes in either the total cellular ATP content or the adenosine diphosphate (ADP)/ATP ratio within 2 days of AOA treatment (Fig. 1D). However, prolonged treatment with AOA for 4 to 6 days significantly decreased the cellular ATP level and increased the ADP/ATP ratio, which indicates that sustained inhibition of glutamine-dependent anaplerosis with AOA may lead to impaired mitochondrial function, including ATP production. Together, these results implicate that the short period (24 h) of AOA exposure-induced mTORC1 inactivation was independent of the change of cellular energy status.

**Fig. 2.**
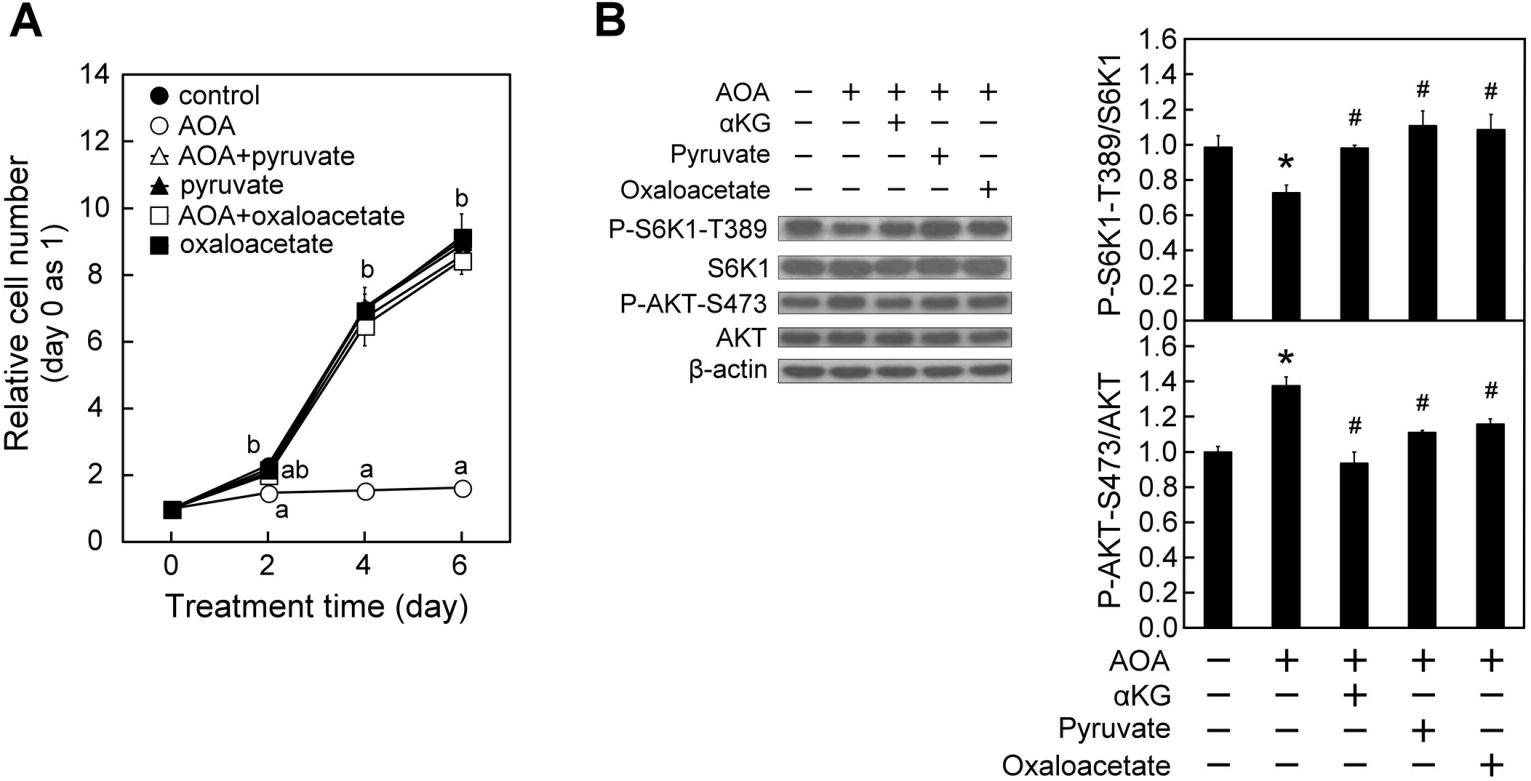
**Anaplerotic factors pyruvate or oxaloacetate, similar to αKG, prevent AOA-induced effects on cell proliferation, mTORC1 and mTORC2 activity in WI38 cells.** WI38 cells were treated with vehicle or AOA in the absence or presence of 3 mM pyruvate, oxaloacetate or 5 mM αKG for the indicated time-period with medium changed at 2-day intervals. (A) Effect of anaplerotic factors on AOA-induced inhibition of cell proliferation. Cell numbers were assessed and calculated as described in Fig. 1A. (B) Effect of anaplerotic factors on AOA modulation of mTORC signaling (*n*=3). After 24-h treatment, cell lysates were prepared and analyzed for the activation status of mTORC1 and mTORC2 respectively indicated by P-S6K1-T389/S6K1 and P-AKT-S473/AKT as described in Fig. 1B. All quantitative data are expressed as the mean±s.e.m. (*n*=3) of three independent experiments. Different lowercase letters indicate significant difference among treatment groups at the same time-point (*P*<0.05). * and # designate a significant difference compared with the respective vehicle control and AOA group, respectively (*P*<0.05).

To further assure the role of glutamine-dependent anaplerosis in regulating mTOR signaling, we examined the effect of two other anaplerotic factors, pyruvate and oxaloacetate (DeBerardinis et al., 2008; Owen et al., 2002), on AOA modulation of cell proliferation and mTORC activity. Pyruvate and oxaloacetate could enter cells respectively through monocarboxylate transporters (MCTs) and NaDCs (Kekuda et al., 1999; Liu et al., 2010). As shown in Fig. 2A, simultaneous supplementation with either pyruvate or oxaloacetate blocked the AOA-induced repression of cell proliferation. In addition, administration of pyruvate or oxaloacetate, like αKG, markedly overturned the AOA-induced mTORC1 inactivation and mTORC2 activation in WI38 cells as indicated by an increase in P-S6K1-T389/S6K1 and a decrease in P-AKT-S473/AKT, respectively (Fig. 2B). These results together support that the anaplerotic entry of a carbon source into the TCA cycle is necessary for mTORC1 activation and mTORC2 attenuation in WI38 cells.

### Sustained inhibition of glutamine-dependent anaplerosis with AOA triggers cellular senescence in WI38 cells that may critically involve p16^INK4A^

**Fig. 3.**
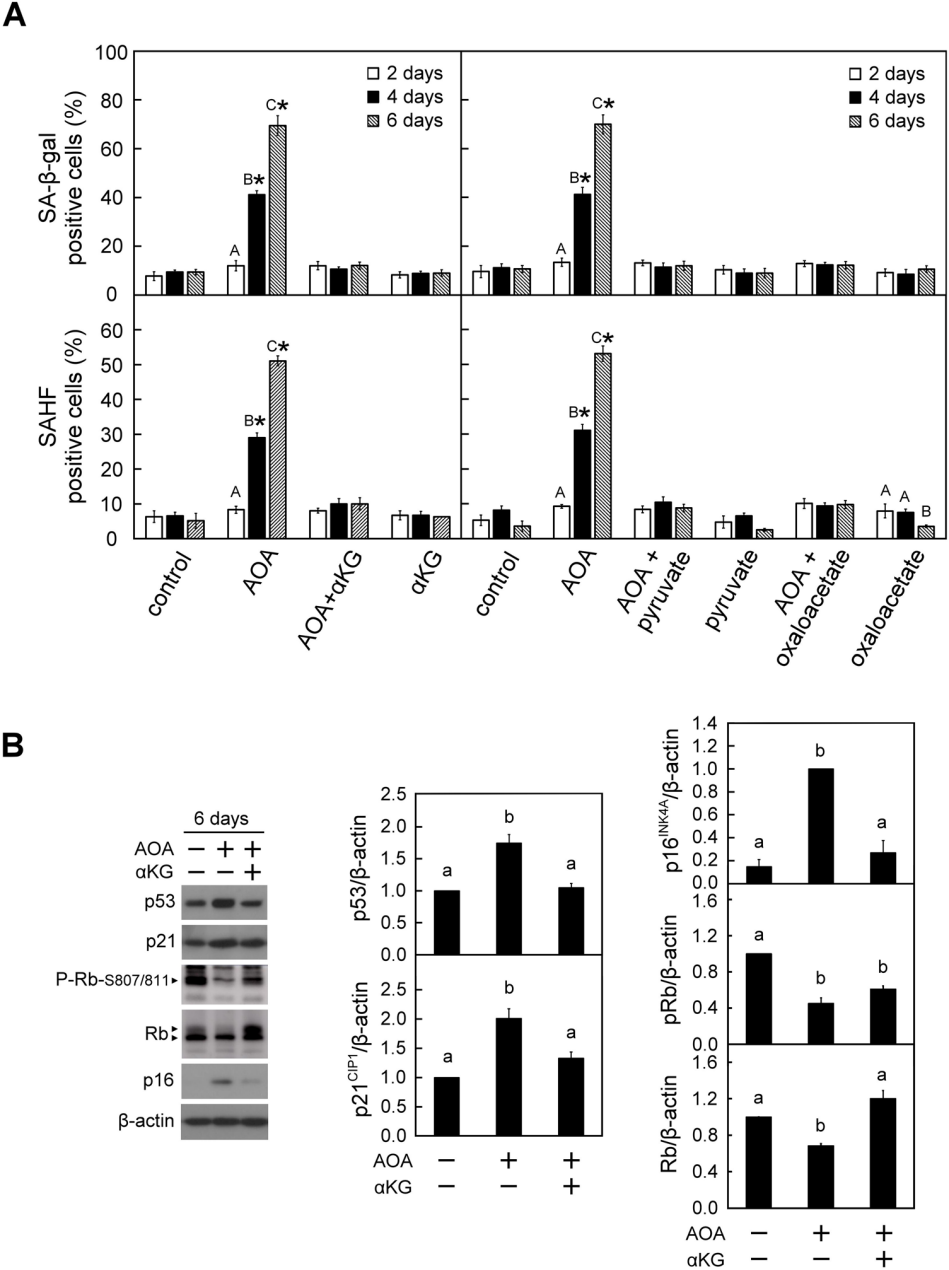
**Prolonged blockade of glutamine-dependent anaplerosis with AOA triggered cellular senescence in WI38 cells.** WI38 cells were treated with vehicle or AOA in the absence or presence of anaplerotic factors αKG, pyruvate or oxaloacetate for the indicated time-period as described in Fig. 1A. (A) Effect of AOA on cellular senescence. The senescent cells were assessed using the SA-β-gal and SAHF staining assays. Upper panel: SA-β-gal positive cells were counted in at least 10 microscopic fields in each of the triplicate cultures of all treatment groups. The percentage of SA-β-gal positive cells was calculated relative to the total cell number in the counted fields. Lower panel: A total of 200 cells from each of the indicated treatment samples were examined for SAHF formation. The percentage of SAHF-positive cells was calculated relative to the total cell number in the counted fields. (B) Effect of AOA on senescence-inducing regulators. After treatment for 6 days, cell lysates were prepared and analyzed by immunoblotting and densitometry analysis for p53, p21^CIP1^, Rb, P-Rb-S807/811 and p16^INK4A^, β-actin served as a loading control. The arrowheads show the indicated antibody recognized specific signals. All quantitative data are expressed as the mean±s.e.m. (*n*=3) of three independent experiments. Different uppercase letters indicate significant difference of the same treatment group at different time-points (*P*<0.05). Asterisk (*) designates a significant difference compared with the respective vehicle control at the same time-point (*P*<0.05). Different lowercase letters indicate significant difference among treatment groups (*P*<0.05).

Perturbation in the homeostasis of mitochondrial metabolites has been suggested to be a critical factor contributing to cellular senescence (Borradaile and Pickering, 2009; Hashizume et al., 2015; Ho et al., 2009; Jiang et al., 2013; Kaplon et al., 2013; Lee et al., 2012; Sahin and DePinho, 2012; van der Veer et al., 2007). The above results (Figs 1 and 2) show that sustained inhibition of glutamine-dependent anaplerosis led to cell cycle arrest and energy depletion. We therefore were intrigued to understand whether prolonged exposure to AOA triggered cellular senescence and the associated molecular mechanism. To identify senescent cells, the WI38 cells were stained with senescence-associated markers, including SA-β-gal activity, SAHF and H3K9Me3 (Narita et al., 2003; Zhang et al., 2007). Both the proportion of SA-β-gal- and SAHF-positive cells were evidently increased after 4 to 6 days of AOA treatment, and this was completely suppressed by simultaneously providing 5 mM of αKG (Fig. 3A, left panel). A similar result was observed for the senescence-associated molecular marker trimethylated histone 3-K9 (H3K9Me3) (Fig. S3). Furthermore, supplementation with two other anaplerotic factors, pyruvate or oxaloacetate, also suppressed the AOA-induced increases in the proportion of SA-β-gal- and SAHF-positive cells (Fig. 3A, right panel).

The p53-p21^CIP1^ and p16^INK4A^ pathways have been reported to be canonical signaling pathways that are involved in cell cycle arrest and senescence (Ben-Porath and Weinberg, 2005). We were interested to explore whether these two are the potential regulatory pathways involved in the AOA-induced cellular senescence. We found that WI38 cells exposed to AOA for 6 days had increased protein levels of p53, p21^CIP1^ and p16^INK4A^, and decreased the level of Rb and P-Rb-S807/811; furthermore, these changes were mitigated by concomitant supplementation with αKG (Fig. 3B).

**Fig. 4.**
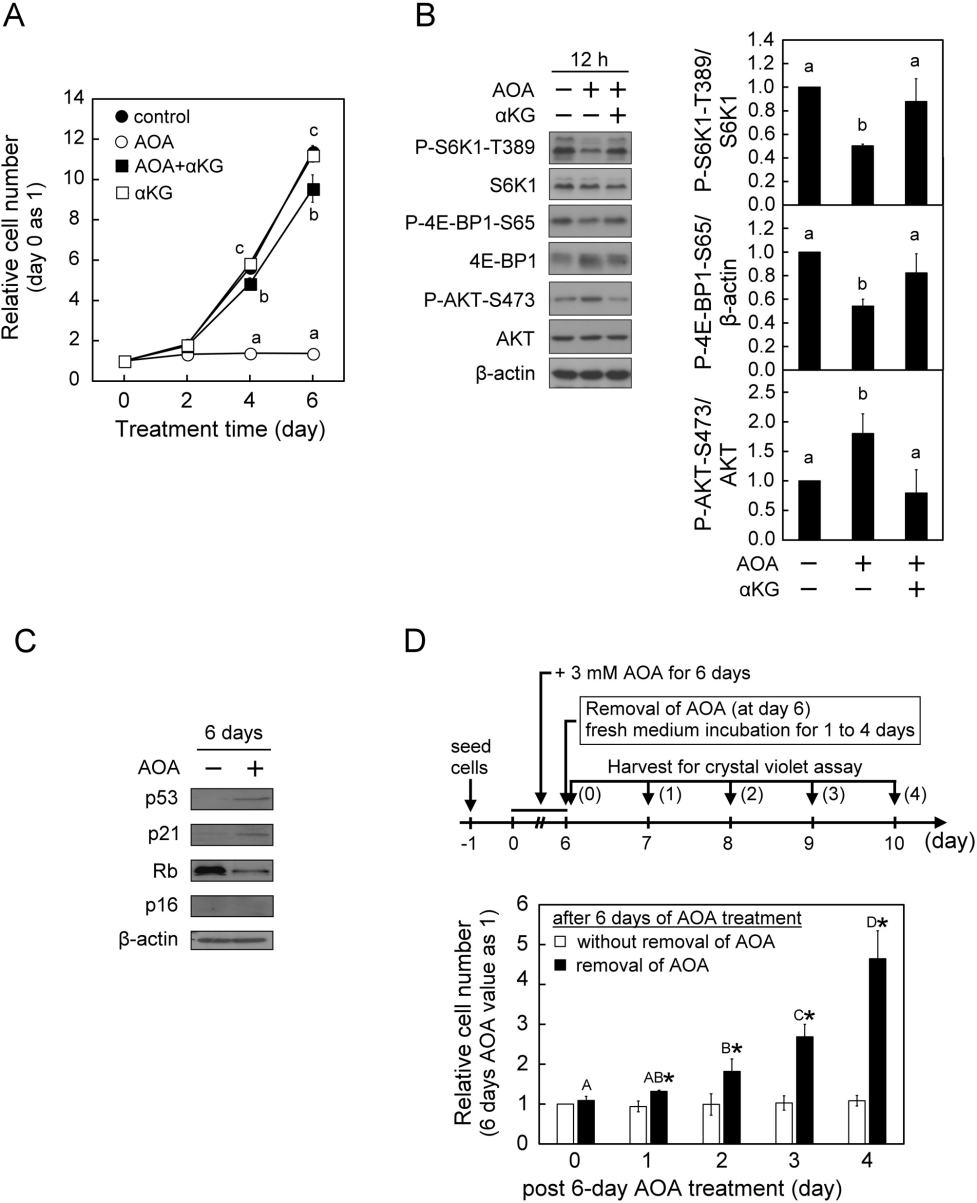
**AOA treatment leads to inhibition of proliferation, mTORC1 inhibition−mTORC2 activation, but not cellular senescence in p16^INK4A^-deficent U2OS cells.** U2OS cells were treated with vehicle or 3 mM AOA in the absence or presence of 5 mM αKG for the indicated time-periods with medium changed at 2-day intervals. (A) Effect of AOA on cell proliferation. Cell numbers were assessed and calculated as described in Fig. 1A. (B) Effect of AOA on mTORC signaling. After 12 h of treatment, cell lysates were prepared and analyzed for the activation status of mTORC1 and mTORC2, respectively, indicated by P-S6K1-T389/S6K1, P-4E-BP1-S65/β-actin and P-AKT-S473/AKT as described in Fig. 1B. (C) Effect of AOA on senescence-inducing regulators. After treatment for 6 days, cell lysates were prepared and analyzed by immunoblotting and densitometry analysis for Rb, p53, p21^CIP1^ and p16^INK4A^, β-actin served as a loading control. (D) Effect of AOA removal on cell proliferation. U2OS cells were treated with 3 mM AOA for 6 days, followed by culture in fresh medium with or without AOA for an additional 4 days. Cell numbers were assessed using Crystal Violet assay, and the relative cell numbers were calculated by normalizing against the value of day 0 of post-6 day AOA treatment. All quantitative data are expressed as the mean±s.e.m. (*n*=3) of three independent experiments, and different lowercase letters indicate significant difference among treatment groups at the same time-point (*P*<0.05). Different uppercase letters indicate significant difference of the same treatment group at different time-points (*P*<0.05). Asterisk (*) designates a significant difference compared with the respective vehicle control (*P*<0.05).

**Fig. 5.**
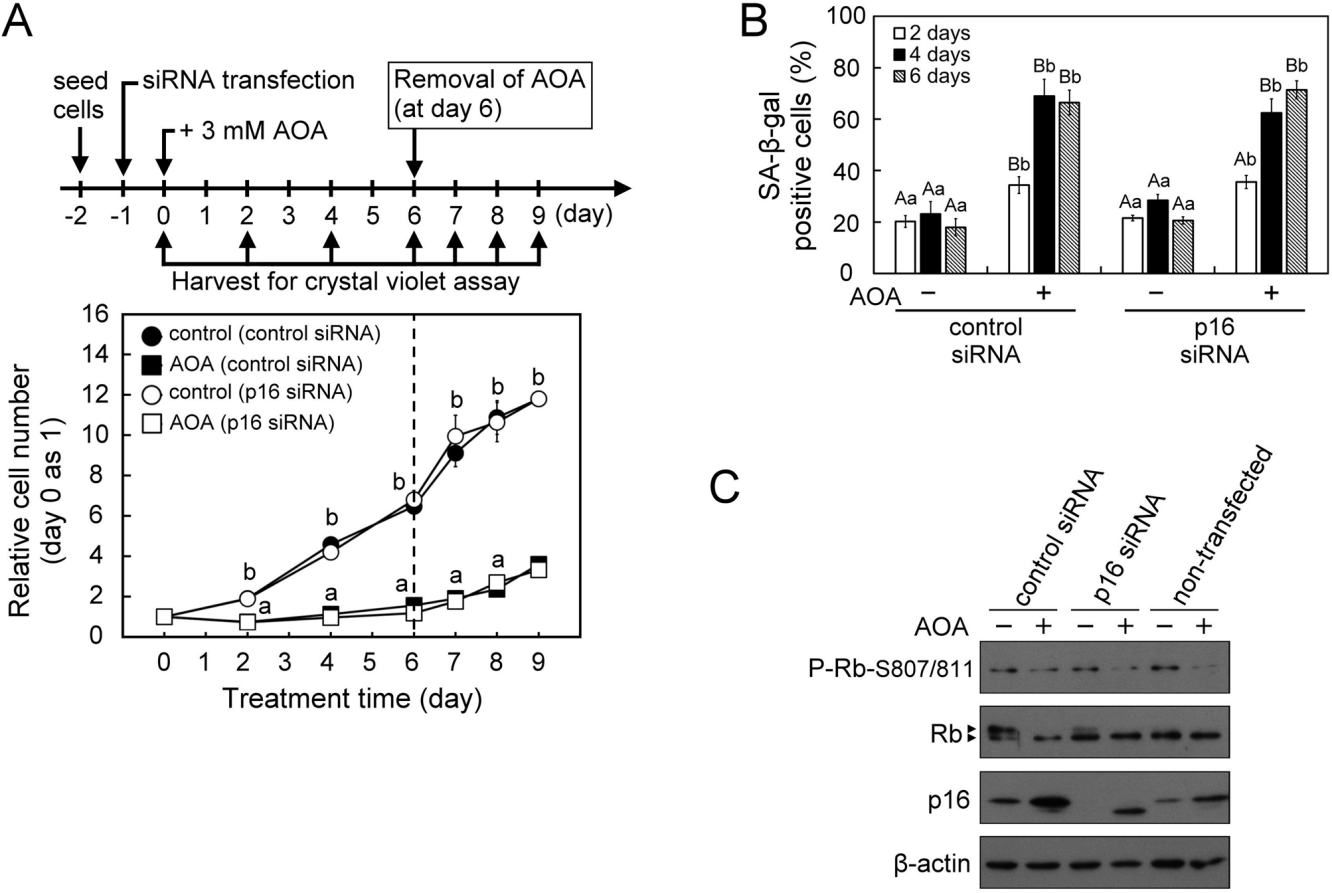
**AOA treatment leads to inhibition of proliferation, increase of p16 antibody-reactive p12 and cellular senescence in p16^INK4A^-knockdown WI38 cells.** Cells were first transfected with p16^INK4A^ siRNA or control siRNA for 24 h, and then treated with vehicle or 3 mM AOA in fresh medium for the indicated time-periods with medium changed at 2-day intervals. (A) Effect of 6-day AOA treatment and 3-day AOA removal on cell proliferation. The relative cell numbers were calculated by normalizing against the value of day 0. After 6 days of AOA treatment, cells were refreshed with AOA-free growth medium and incubated for an additional 3 days. (B) Effect of AOA on cellular senescence. The senescent cells were assessed using the SA-β-gal staining assay. SA-β-gal positive cells were counted in at least five microscopic fields each of the triplicate cultures of all treatment groups. The percentage of SA-β-gal positive cells was calculated relative to the total cell number (DAPI-stained positive cells) in the counted fields. (C) Effect of AOA on the senescence-inducing regulator p16^INK4A^−Rb pathway. At the end of 6-day AOA treatment, cell lysates were prepared for immunoblotting and densitometry analysis of p16^INK4A^, Rb and P-Rb-S807/811 with β-actin served as a loading control. All quantitative data are expressed as the mean±s.e.m. (*n*=5) of two independent experiments. The arrowheads show the indicated antibody recognized specific signals. Different lowercase letters indicate significant difference among treatment groups at the same time-point (*P*<0.05). Different uppercase letters indicate significant difference of the same treatment group at different time-points (*P*<0.05).

To further investigate the specific role of p16^INK4A^ in the induction of cellular senescence, we first examined the effect of AOA on cell growth and cellular senescence in the p16^INK4A^-deficient U2OS human osteosarcoma cell line. Similar to the observations in WI38 cells, treatment of U2OS cells with AOA also led to inhibition of proliferation, as well as mTORC1 inactivation and mTORC2 activation during the 6-day culture period (Fig. 4A,B). Interestingly, while prolonged (6 days) AOA treatment of U2OS cells increased the protein levels of p53 and p21^CIP1^ (Fig. 4C) like those observed in normal human fibroblast WI38 cells, no significant changes were observed in the SA-β-Gal activity and appearance of SAHF (data not shown). In U2OS cells, we also observed a distinct finding that the prolonged (6 days) AOA exposure-induced inhibition of proliferation was promptly reverted after removal of the AOA from the medium (Fig. 4D). The different effects of AOA on the induction of cellular senescence in WI38 and p16^INK4A^-deficient U2OS cells imply that the p16^INK4A^ pathway plays a critical role in triggering cellular senescence under conditions where the inhibition of glutamine-dependent anaplerosis is sustained. Next, to understand the role of p16^INK4A^ in AOA-induced cell growth arrest and senescence in normal proliferation-active cells, a siRNA knockdown strategy was used in WI38 cells. Consistent with the finding in p16^INK4A^-deficient U2OS cells (Fig. 4A,D), treatment with AOA also suppressed proliferation in p16^INK4A^-knockdown WI38 cells, and the growth-arrested cells resumed proliferation activity after the removal of AOA (Fig. 5A). In contrast to the finding in p16^INK4A^-deficient U2OS cells, sustained AOA treatment (4 to 6 days) still increased senescence-associated SA-β-Gal activity in p16^INK4A^-knockdown WI38 cells, similar to the control siRNA group (Fig. 5B). AOA treatment also decreased the level of P-Rb-S807/811 in p16^INK4A^-knockdown WI38 cells as in the control cells (Fig. 5C). Interestingly, we noticed in p16^INK4A^-knockdown WI38 cells that AOA treatment clearly induced an increase of an estimated 12-kDa p16^INKA^-specific antibody-reactive protein band, while AOA increased p16^INK4A^ protein level in control-siRNA-treated group just like the non-transfected WI38 cells (Fig. 5C). It is speculated that knockdown of p16^INK4A^ in WI38 cells may be compensated by an increase of the alternative spliced variant form of p16^INK4A^ protein (p12) under AOA disruption of metabolic regulation, and thus still induced senescence process. This awaits further investigation.

### AOA-induced cellular senescence in WI38 cells was independent of ROS signals

**Fig. 6.**
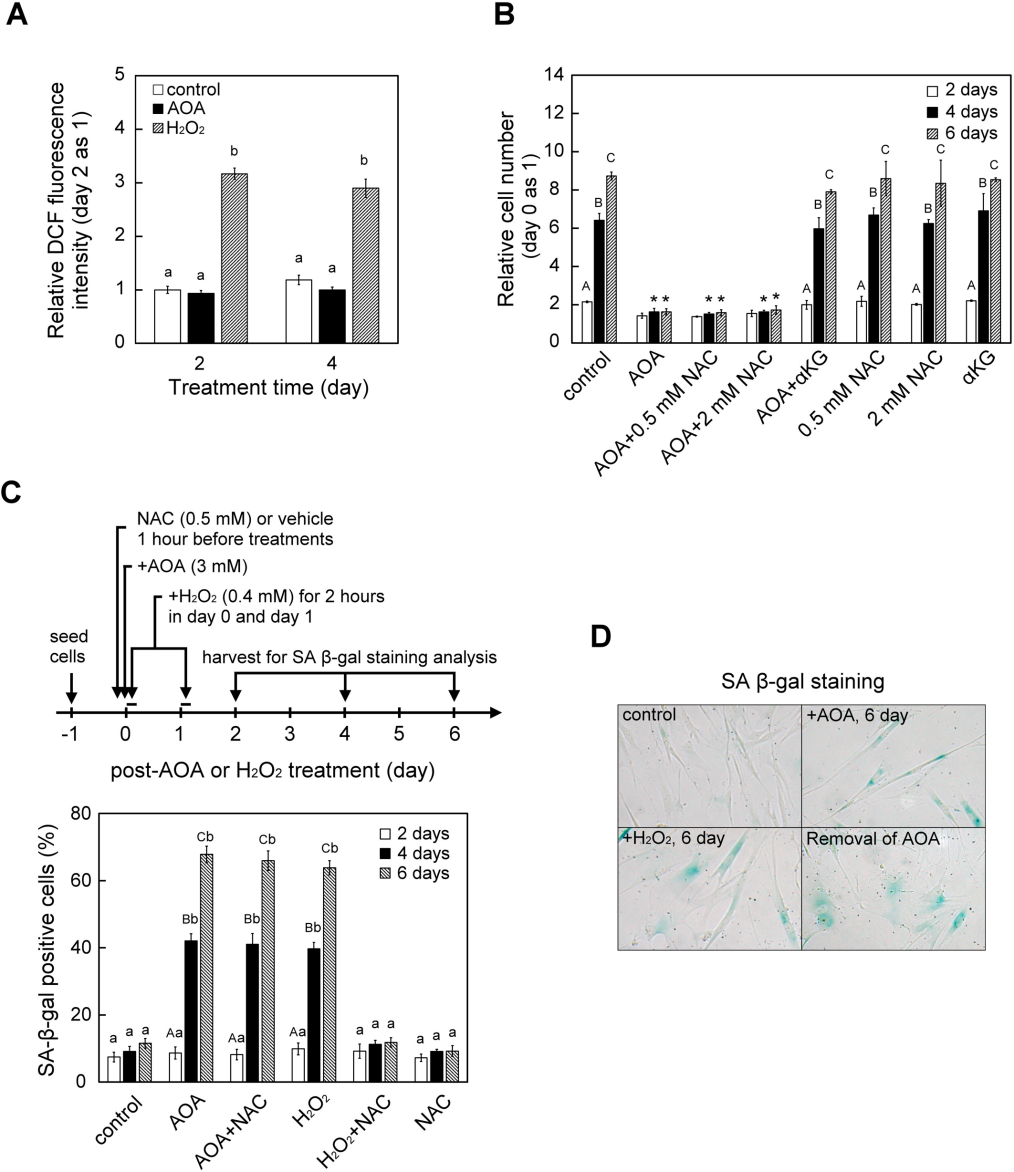
**AOA-induced cell growth arrest and senescence of WI38 cells was not mediated by ROS signaling.** (A) Effect of AOA on cellular ROS level. WI38 cells were treated with 3 mM AOA for 2 and 4 days (*n*=9). As a positive control, cells were exposed to 0.4 mM H_2_O_2_ for 2 h on the first 2 days. At the indicated time-period, cellular content of ROS was determined using DCFH-DA staining as detailed in the Materials and Methods. (B) Effect of ROS scavenger NAC on cell proliferation. Cells were pre-treated with vehicle or NAC for 1 h and then treated with or without 3 mM AOA for the indicated time-periods with medium changed at 2-day intervals. Cell numbers were assessed and calculated as described in Fig. 1A. (C) Effect of NAC on AOA-induced SA-β-gal activity. Cells were pre-treated with vehicle or 0.5 mM NAC for 1 h prior to treatment with AOA or H_2_O_2_ for the indicated time-period. As a positive control, WI38 cells were exposed to 0.4 mM H_2_O_2_ for 2 h on the first 2 days in the absence or presence of NAC. Senescent-like cells were evaluated by measuring SA β-gal activity as described in Fig. 3A. (D) Effect of chronic AOA treatment on cell morphology of WI38 cells. The cells were grown in the absence (control) or presence (+AOA, 6 day) of 3 mM AOA for 6 days, or in the presence of AOA for 6 days followed by removal of the AOA for an additional day (Removal of AOA). The H_2_O_2_-induced senescent WI38 cells (+H_2_O_2_, 6 day) serving as a reference became enlarged and flattened, which is a documented typical senescent morphology. All quantitative data are expressed as the mean±s.e.m. (*n*=3) of three independent experiments. Different lowercase letters indicate significant difference among treatment groups at the same time-point (*P*<0.05). Different uppercase letters indicate significant difference of the same treatment group at different time-points (*P*<0.05). Asterisk (*) designates a significant difference compared with the respective vehicle control at the same time-point (*P*<0.05).

Mitochondrion-derived reactive oxygen species (ROS) have been reported to inhibit cell proliferation and induce cellular senescence in many cell types including human fibroblasts (Chen and Ames, 1994; von Zglinicki et al., 1995). Because glutamine-dependent anaplerosis is critical to balancing the concentration of TCA cycle intermediates and maintaining mitochondrial homeostasis, thus sustained inhibition of glutamine-dependent anaplerosis has the potential to trigger ROS production. We first examined whether AOA exposure affects ROS level in WI38 cells using DCF detection assay, and found that there were no obvious changes in ROS level following AOA treatment for 2 to 4 days, while cells exposed to hydrogen peroxide (H_2_O_2_) serving as a control had increased ROS level (Fig. 6A). We next used ROS scavenger N-acetyl-L-cysteine (NAC) to further examine whether ROS are involved in AOA-induced growth arrest and cellular senescence. WI38 cells were pre-treated with 500 μM NAC for 1 h before exposure to 400 μM H_2_O_2_ (as a control) or 3 mM AOA, and the cells were stained for SA-β-gal activity 2, 4 and 6 days later. Similar to the AOA effect, treatment with H_2_O_2_ also increased the proportion of SA-β-gal-positive cells 4 and 6 days later; moreover, pretreatment with NAC did block the H_2_O_2_-induced effect (Fig. 6B; H_2_O_2_ versus H_2_O_2_+NAC), whereas pretreatment with NAC did not affect the AOA-induced increased proportion of cell growth arrest and SA-β-gal-positive cells (Fig. 6B,C; AOA versus AOA+NAC) in WI38 cells.

An enlarged and flattened morphology is a typical documented characteristic of senescent fibroblasts (Straface et al., 2007). Interestingly, we observed AOA-induced senescent cells exhibited a different elongated and non-spreading morphology (Fig. 6D; AOA 6 days). To investigate whether this AOA-induced morphological change was associated with senescence response, AOA-induced senescent WI38 cells were obtained after 6 days of treatment, and then senescent cells were incubated in AOA-free medium for an additional day. Notably, 1 day after removal of AOA, the AOA-induced senescent WI38 cells became morphologically flattened and enlarged (spreading) with cell shapes similar to those of the H_2_O_2_-induced senescent WI38 cells (Fig. 6D; removal of AOA versus H_2_O_2_ 6 days). This observation implied that the AOA-induced morphological changes were independent of the maintenance of cellular senescence.

## DISCUSSION

Glutamine-dependent anaplerosis plays a critical role in proliferating cells to support the proper operation of the mitochondrial TCA cycle to provide biosynthetic precursors and energy (DeBerardinis et al., 2008; Lunt and Vander Heiden, 2011; Yuneva et al., 2007). This study was initiated to explore how glutamine-dependent anaplerosis contributes to cell fate determination (cell proliferation and senescence) by employing AOA, an inhibitor of glutamate-dependent aminotransferase, to block glutamate conversion to αKG (Kaadige et al., 2009; Wise et al., 2008; Wise and Thompson, 2010), and supplement with anaplerotic factor (αKG, pyruvate or oxaloacetate) to confirm the specificity of AOA on blocking glutamine-dependent anaplerosis. We unveil that in WI38 normal human embryonic fibroblasts, glutamine-dependent anaplerosis is essential to cell growth and closely associated with mTORC1 activation and mTORC2 inactivation, and impedes cellular senescence particularly associated with p16^INK4A^.

### What is the relation between glutamine-dependent anaplerosis and mTORC1/2 activity and cell growth?

In normal human embryonic fibroblasts (WI38 cells), inhibition of glutamine-dependent anaplerosis with AOA first led to mTORC1 inactivation and mTORC2 activation within 12 to 24 h, and cell growth arrest from day 2 to 6; notably, supplement with anaplerotic factors (αKG, pyruvate or oxaloacetate) blocked these AOA-induced effects (Figs 1 and 2). This implicates that continuous support of anaplerotic entry of carbon backbone into the TCA cycle is required to modulate proper activity of mTORC1 and mTORC2, and commit cell growth and cell cycle progression.

Previous studies mostly using starvation-refeeding protocols have indicated that mTORC1 is the most important regulator of cell growth and proliferation by coordinating the biosynthetic activity with nutrient availability including amino acids (Chen et al., 2014; Yao et al., 2017; Ye et al., 2015). Considering the situation when mammalian cells are not in a nutrient-deficient environment, the crucial issue regarding mTORC1 regulation of cell growth may likely be the control of metabolic pathways rather than nutrient availability itself. This concept is supported by our current study, as anaplerotic factors (αKG, pyruvate or oxaloacetate) could repress the AOA-induced mTORC1 inactivation (Figs 1B and 2B), and the effect of αKG was inhibited by mTORC1 inhibitor rapamycin (Fig. 1C). In addition, a recent study reported that anaplerotic entry of αKG is required for mTORC1 activity by serving as a fuel for the TCA cycle-mediated production of ATP to stabilize the mTORC1/2 chaperone, TTT-RUVBL1/2 complex, as inactivation of ATP-sensitive TTT-RUVBL1/2 complex causes instability of both mTORC1 and mTORC2 (Kim et al., 2013). Our work in WI38 normal human embryonic fibroblasts, however, shows that AOA exposure caused mTORC1 inactivation and mTORC2 activation within 24 h (Figs 1B,C and 2B) when intracellular ATP levels and the ADP/ATP ratio were not affected (Fig. 1D), suggesting that the AOA effect on the activity of mTORC1/2 and cell growth is most likely not mediated by ATP-dependent stabilization of mTORC1/2 or ATP depletion. Furthermore, we observed that AOA exposure induces the opposite effect on mTORC1 (inactivation) and mTORC2 (activation) in normal human embryonic fibroblast WI38 cells (Figs 1B,C and 2B). This may likely be due to the negative feedback regulatory loop of mTORC1 towards to mTORC2 (Julien et al., 2010; Liu et al., 2014; Sabatini, 2006).

On the other hand, although glutamine may serve as carbon and nitrogen sources in proliferating cells, our current results (Figs 1A and 2A) suggest that the carbon backbone of glutamine is indispensable for G_1_ progression in normal cells. Consistent with our observations, recent studies have reported that glutaminase-1, which catalyzes the first step of glutaminolysis, is required for proliferating cells to progress through the restriction point of the G_1_ phase by maintaining a high abundance of TCA intermediates (Est; ez-Garc; et al., 2014), and that metabolic pathways influence cell cycle progression (Almeida et al., 2010; Duan and Pagano, 2011). Interestingly, growing evidence indicates there is a metabolic checkpoint in controlling cell cycle progression from G_1_ to the S phase to ensure adequate metabolic activity and sufficient building blocks before committing to duplicate the genome (Kalucka et al., 2015; Mohrin and Chen, 2016). Therefore, these observations implicate that sustained glutamine-dependent anaplerosis activity is essential for proper activity of mTORC1/2, and is linked to cell cycle progression to make sure there is sufficient availability of glutamine for cell growth.

### What is the critical factor that means restraint of glutamine-dependent anaplerosis initiates senescence-like response in proliferating cells?

We showed, in WI38 normal human embryonic fibroblasts, that prolonged AOA treatment triggers a senescence-like response (within day 4 to 6) as indicated by increased percentages of SA-β-gal-positive, SAHF-positive and H3K9Me3-positive cells, as well as elevated protein levels of p53, p21^CIP1^ and p16^INK4A^ and decreased protein levels of Rb and P-Rb-S507/811; while supplementation with anaplerotic factor (αKG, pyruvate or oxaloacetate) prevented AOA-induced senescence (Fig. 3; Fig. S3). This implicates that prolonged constraint of anaplerotic supply of carbon backbone ultimately triggers a cellular senescence-like response.

Glutamine-dependent anaplerosis is essential for the maintenance of mitochondrial functions (DeBerardinis et al., 2007; Fan et al., 2013; Strohecker and White, 2014). Since mitochondrial dysfunction may generate ROS, which can cause a deleterious effect to cell proliferation, it is proposed that excessive mitochondrial ROS plays a critical role to trigger cellular senescence (Jiang et al., 2013; Moiseeva et al., 2009; Velarde et al., 2012). However, our results show that AOA did alter cellular ROS levels, and that AOA-induced senescence and proliferation were not affected by the ROS scavenger NAC (Fig. 6). These observations suggest that ROS signal is not critically involved in sustained inhibition of glutamine-dependent anaplerosis-induced senescence in WI38 human embryonic fibroblasts. Many other studies have also shown that mitochondria-derived ROS may not necessarily be the primary cause of senescence and aging. For example, in human fibroblasts, sustained pharmacological inhibition of OXPHOS with antimycin A or oligomycin induces cellular senescence, but this is not due to increased ROS levels (Stockl et al., 2006). In the mouse model, mitochondrial dysfunction-induced ageing was not associated with increased oxidative stress (Kujoth et al., 2005; Trifunovic et al., 2005). Moreover, an empirical mathematical model study has shown that increased mitochondrial ROS in replicative senescent cells is a consequence of the senescence phenotype rather than the cause (Lawless et al., 2012). These observations implicate that in addition to mitochondrion-derived ROS, there are other factors involved in triggering mitochondrial dysfunction-induced senescence.

On the other hand, the induction of cellular senescence has been demonstrated to rely on the activation of p53-p21^CIP1^ and/or p16^INK4A^ pathways in normal human cells (Beausejour et al., 2003; Qian and Chen, 2013). We found that sustained inhibition of glutamine-dependent anaplerosis with AOA resulted in increased protein levels of p53-p21^CIP1^ (day 3 to 6) and p16^INK4A^ (day 6) in WI38 human embryonic fibroblasts; importantly, supplement with αKG prevented AOA-induced changes of these three proteins (Fig. 3B; Fig. S4). Similar to replicative senescence of diploid human fibroblasts (Alcorta et al., 1996; Stein et al., 1999), AOA-induced senescence of WI38 cells also caused sequential increases of these proteins with p53-p21^CIP1^-mediated cell cycle arrest as the early reversible event of senescence response, and p16^INK4A^-mediated the late irreversible event of senescent arrest. Moreover, Rb protein has also been reported to gradually decline in replicative senescent fibroblasts (Helmbold et al., 2009), while the role of Rb in senescence needs further investigation. In addition, it has been shown that senescent human fibroblasts display decreased ATP level, and increased AMP/ATP ratio and AMPK activity; furthermore, induction of AMPK activity can cause cellular senescence (Wang et al., 2003). AMPK is considered a central regulator during cell response to energy stress (Chang et al., 2010; Jones et al., 2005; Wiley et al., 2016). The current study found that prolonged AOA exposure (day 4 to 6) led to reduced ATP level and increased ADP/ATP ratio, and cellular senescence-like response in WI-38 cells (Figs 1D and 3A; Fig. S3); whether AMPK activity is involved in this process requires further investigation. These observations implicate that senescence response induced by prolonged restraint of glutamine-dependent anaplerosis resembles replicative senescence response. In addition, supplementation with αKG at day 0 to 3 after AOA exposure almost completely repressed the AOA-induced SA-β-gal activity, while αKG supplemented at day 4 to 5 only partially attenuated the AOA effect (Fig. S5). Our current findings support that AOA-induced senescence in WI38 human embryonic fibroblasts may contain two phases, a reversible cell cycle arrest and ultimately an irreversible senescent arrest.

In p16^INK4A^-deficient U2OS cells, we found that inhibition of glutamine-dependent anaplerosis with AOA also caused mTORC1 inactivation, mTORC2 activation, increased protein levels of p53 and p21^CIP1^, and inhibited cell proliferation that could be resumed after removal of AOA (Fig. 4A–D), but failed to induce senescence-associated SA-β-gal activity (data not shown). While in p16^INK4A^-knockdown WI38 cells, AOA exposure also similarly inhibited cell proliferation (Fig. 5A). In contrast to p16-deficient U2OS cells, sustained AOA treatment still induced senescence-associated SA-β-gal activity like that in control WI38 cells (Fig. 5B), and this was accompanied by the induction of p16^INK4A^ antibody-reactive p12 (Fig. 5C). It is speculated that knockdown of p16^INK4A^ in WI38 cells may be compensated by an increase of the alternative spliced variant form of p16^INK4A^ protein (p12) under AOA disruption of metabolic regulation, and thus still induced senescence process. Together, these observations implicate that p16^INK4A^ is crucial to establish AOA-induced irreversible senescent arrest. Of note, we observed that AOA-induced mTORC2 activation as indicated by elevated phosphorylation of AKT at Ser473, compared with untreated control (Figs 1B and 2B). It has been reported that AKT plays a role in induction of senescence-like response (Miyauchi et al., 2004; Nogueira et al., 2008; Taylor et al., 2011), indicating that AOA-induced activation of mTORC2 may contribute to the cell fate determination of WI38 cells to senescence-like response. Moreover, since mTORC2 has been reported to control actin polymerization (Jacinto et al., 2004; Saci et al., 2011), AOA-induced elongated and non-spreading morphology of WI38 cells may be due to the aberrant mTORC2 activity.

Our current study suggests that the status of glutamine-dependent anaplerosis plays a critical role in growing cells to coordinate the glutamine metabolism and mTORC1/2 activity, and is linked to the cell-cycle checkpoint to make sure intracellular sufficiency of metabolites for cell growth. Moreover, increasing evidence implicates that cellular metabolism correlated with the balance of mitochondrial metabolites plays a role in cellular senescence (Borradaile and Pickering, 2009; Hashizume et al., 2015; Ho et al., 2009; Jiang et al., 2013; Kaplon et al., 2013; Lee et al., 2012; van der Veer et al., 2007). We also provide evidence that persistent restraint of glutamine-dependent anaplerosis is a critical factor to contribute to cellular senescence and in a ROS-independent manner. Together, the present study provides a mechanism by which proliferating cells coordinate the intracellular nutrient sufficiency and mTOR signaling, and links to senescence-like response as a fail-safe mechanism to limit deleterious effect for organismal homeostasis.

## MATERIALS AND METHODS

### Materials

AOA, H_2_O_2_, NAC, αKG, dimethylα-ketoglutarate (dmαKG), sodium pyruvate, oxaloacetate and 2′,7′-dichlorodihydrofluorescein diacetate (DCFDA) were purchased from Sigma-Aldrich. Rapamycin and Torin1 were purchased from Tocris Bioscience (Minneapolis, USA). Stock solutions of AOA (0.5 M), αKG (0.5 M), pyruvate (0.5 M), oxaloacetate (0.5 M) and NAC (0.5 M) were prepared in ddH_2_O, sterilized by filtration and then stored as aliquots at −20°C. Rapamycin and Torin1 were dissolved initially in DMSO to 1 mM and 750 µM stock solutions, respectively. DCFDA was freshly prepared in DMSO at 5 mM. The antibodies against S6K1 (#9202), P-S6K-T389 (#9205), 4E-BP1 (#9452), P-4E-BP1-S65 (#9451), AKT (#9272) and P-AKT-S473 (#9271) were purchased from Cell Signaling Technology; the antibodies against pRb (#IF8), p53 (#DO1), p21^CIP1^ (#F5) and p16^INK4A^ (#H156) were from Santa Cruz Biotechnology; another anti-p16 (ab81278) and anti-H3K9Me3 (ab8898) antibodies were from Abcam (Cambridge, UK); anti-β-actin was from Sigma-Aldrich; and all HRP-conjugated secondary antibodies were from Pierce Thermo Fisher Scientific.

### Cell culture and treatments

The WI38 normal human embryonic lung fibroblast cell line and the U2OS human osteosarcoma cell line (ATCC, Manassas, USA) were grown in minimum essential medium (MEM; Sigma-Aldrich) supplemented with 10% (v/v) fetal bovine serum (FBS; Gibco Life Technologies), 2 mM L-glutamine, 1 mM sodium pyruvate and antibiotic (100 U ml^−1^ penicillin and 100 μg ml^−1^ streptomycin) at 37°C in 5% CO_2_. For the AOA treatments, the cells were chronically exposed to vehicle or 3 mM AOA, and the medium containing without or with AOA was refreshed every 2 days. For the combination treatments with anaplerotic factors (αKG, pyruvate or oxaloacetate) and AOA, the cells were chronically exposed to 3 mM AOA in the absence or presence of 5 mM αKG, 3 mM pyruvate or 3 mM oxaloacetate for various time-periods. The cellular anaplerotic factors αKG, oxaloacetate and pyruvate could enter cells respectively through secondary active transporters SLC13 family NaDCs and MCTs (Kekuda et al., 1999; Liu et al., 2010; Pajor, 2014). In addition, we compared the effectiveness of αKG and a cell permeable analog dmαKG in alleviating AOA-induced growth arrest. Results are shown in Fig. S1. Both αKG and dmαKG dose-dependently (0.2 to 5 mM) blocked AOA-induced growth arrest in WI38 cells during the 6-day culture period (effectiveness: αKG>dmαKG), while they alone had no influence on cell growth. We also observed that WI38 cells treated with high-dose (25 mM) αKG alone had reduced growth rate, yet it still could block AOA effect though less effectively than that of 5 mM αKG. On the other hand, WI38 cells given a high dose (25 mM) of dmαKG alone or together with AOA, which caused mild cell loss during the 6-day culture period. Whether this was in part due to the documented pseudohypoxia effect of dmαKG through stabilization of HIF-1α (Hou et al., 2014) awaits further investigation. For the H_2_O_2_ treatment, cells were treated with 400 μM H_2_O_2_ for 2 h only on day 0 and day 1, and then cells were incubated in growth medium without H_2_O_2_ for an additional time-period as indicated. For co-treatment with H_2_O_2_ and NAC, after a 15-min pre-treatment with vehicle or 500 μM NAC, the cells were exposed to 400 μM H_2_O_2_ as described above in the absence or continued presence of 500 μM NAC; after 2-h H_2_O_2_ exposure the medium was replaced with growth medium containing NAC.

### Knockdown of p16^INK4A^ in WI38 cells

WI38 cells were transfected with p16^INK4A^ siRNA (#6598) or non-targeting siRNA (control siRNA; #6568) (both Cell Signaling Technology) by the RNAiMAX reagent (Life Technologies) according to the manufacturer's instructions. Briefly, 3.6×10^4^ cells per well or 1.8×10^5^ cells per dish were seeded in antibiotic-free growth medium a day before transfection. The siRNA and lipofectamine RNAiMAX were diluted separately with MEM for 5 min and then mixed together at room temperature for 20 min. Then, the siRNA-liposome complex was added into wells with a final concentration of 50 nM siRNA. The cells were then subjected to treatment for 3 to 6 days after transfection and cell lysates were prepared for immunoblotting analysis.

### Cell extraction and western blotting

Total cell extracts were prepared with RIPA lysis buffer: 50 mM Tris-HCl (pH 7.4), 2 mM EDTA, 150 mM NaCl, 1% NP-40, 0.5% sodium-deoxycholate, 0.1% SDS, 50 mM NaF, 1 mM Na_3_VO_4_, and a protease inhibitor cocktail (Roche Molecular Biochemicals). The soluble fractions of the cell lysates were isolated by centrifugation at 14,000×**g** for 15 min at 4°C. The protein concentration in the resulting supernatant was determined using the Bradford assay (Bio-Rad). For the western blotting, protein samples (20 μg) were boiled in SDS sample buffer, resolved using SDS-PAGE, and transferred to PVDF membranes. The membrane was blocked with 5% non-fat milk, and then sequentially incubated with the primary antibody and HRP-conjugated secondary antibody. The specific protein signals were visualized using enhanced chemiluminescence substrate (Perkin Elmer, Massachusetts, USA) followed by detection using X-ray films or the FluorChem M system (ProteinSimple, San Jose, USA). The density of each detected protein band was quantified using ImageJ software (version 1.49b, National Institutes of Health) and normalized to that of β-actin.

### Cell number assessment

For cell growth experiments, approximately 1.5×10^4^ cells per well were plated in 24-well culture plates, and given various treatments for the indicated time-periods. At the end of culture, cell numbers were assessed using a Crystal Violet assay as previously described (Gillies et al., 1986) with slight modifications. In brief, the cells were rinsed with PBS, fixed in 4% paraformaldehyde for 10 min, and stained with 0.1% Crystal Violet for 30 min at room temperature. The plates were then washed with ddH_2_O, washed out until colorless and air-dried overnight. The Crystal Violet stain was solubilized in 10% acetic acid, and the optical absorbance was measured at 590 nm. Each individual experiment was repeated at least three times.

### Cell cycle analysis

The cells were treated with 3 mM AOA for 48 h, trypsinized and fixed in 70% (v/v) ice-cold ethanol at 20°C overnight. The cells were washed twice with PBS and resuspended in a Propidium Iodide staining solution (25 µg ml^−1^ PI and 50 μg ml^−1^ DNase-free RNase) at 37°C for 30 min in the dark. The stained cells were then analyzed using a flow cytometer (BD FACSAria III, San Jose, USA). At least 10,000 events were recorded per sample. The cell cycle distributions were analyzed using ModFit LT software (Verity Software House, Topsham, USA).

### SA-β-gal staining and SAHF formation assay

SA-β-gal activity was detected as previously described (Dimri et al., 1995) with slight modifications. After treatment, the cells were washed once with PBS, and then fixed (2% formaldehyde/0.2% glutaraldehyde in PBS) for 10 min at room temperature. Then, the cells were stained in freshly prepared SA-β-Gal staining solution [1 mg ml^−1^ X-gal, 40 mM citric acid/sodium phosphate (pH 6.0), 5 mM potassium ferrocyanide, 5 mM potassium ferricyanide, 150 mM NaCl, 2 mM MgCl_2_] for 16 h at 37°C. The plates were then washed twice with PBS and stored in PBS. Before microscopic examination of SA-β-Gal activity, nuclei were stained with DAPI (10 µl ml^−1^) for 10 min. Ten fields of each sample well were photographed using a Zeiss Axiovert100 microscope. The percentages of cells positive for β-gal activity (blue staining) were determined, and calculated as the ratio of SA β-gal-positive cells to the total number of DAPI-stained cell nuclei. Each experiment was performed in triplicate per treatment group, and repeated independently at least three times. For the SAHF assay, the cells were stained with DAPI to visualize the nuclei, and the percentages of SAHF-positive cells with condensed DAPI-stained foci were determined by scoring 200 individual cells for each sample. Three independent experiments were performed.

### Immunofluorescent staining of H3K9Me3

Cells were seeded on gelatin-coated coverslips at least 24 h prior to treatment. After rinse with PBS, cells were fixed in freshly prepared 4% paraformaldehyde for 10 min at 37°C, then washed three times with PBS. Cells were permeabilized with 0.2% Triton X-100 in PBS for 10 min at room temperature, and washed twice in PBS. Then, cells were blocked for 1 h with 3% (w/v) BSA in PBS, and incubated with rabbit anti-H3K9Me3 antibody (1:1000; ab8898, Abcam) or goat IgG at 4°C overnight. Cells then were washed three times with PBS, and incubated with secondary antibody, goat anti-rabbit IgG conjugated with AlexaFluor 488 (1:1000, Molecular Probes-Invitrogen) for 1 h at room temperature. Photographs were taken using a fluorescent microscope (OLYMPUS, BX50).

### Measurement of ATP and ADP

The determination of ATP and ADP was conducted as previously described (Goto et al., 2006) using ApoGlow Assay Kit (Adenylate Nucleotide Ratio Assay; Cambrex, Rockland, USA) according to the manufacturer's instructions. Briefly, the cells were plated in a 24-well plate, and treated as indicated above. At the end of culture, the cells were lysed for 5 min, the lysates were transferred into opaque white 96-well plates and the luminescence intensity was detected using the Molecular Devices F3 microplate reader. Three independent experiments were performed.

### Measurement of ROS

ROS production was measured using the fluorescent probe 2′,7′-dichlorofluorescin diacetate (DCFDA) as previously described (Wu and Yotnda, 2011) and according to the manufacturer's instruction (D6883, Sigma-Aldrich). Briefly, the cells were plated in a 24-well plate, and treated as indicated above. At the end of culture, the conditioned media were removed, and the cells were loaded with 5 µM DCFDA for 30 min at 37°C. The cells were then washed three times in phenol red-free MEM, and then the fluorescence intensity was determined using a Molecular Devices F3 microplate reader with the excitation set at 490 nm and the emission detected at 520 nm.

### Statistics

The quantitative data are expressed as the mean±s.e.m. of at least three independent experiments. All statistical analyses were performed using IBM SPSS statistics software (version 25, Chicago, USA) and GraphPad Prism (version 7.0, La Jolla, USA). Statistical analysis was performed using one-way ANOVA and post-hoc Tukey's test for multiple group comparison, or paired Student's *t-*test for two-group comparison. *P*<0.05 was considered statistically significant in all experiments.

## Supplementary Material

Supplementary information

## References

[BIO038257C1] Alcorta D. A., Xiong Y., Phelps D., Hannon G., Beach D. and Barrett J. C. (1996). Involvement of the cyclin-dependent kinase inhibitor p16 (INK4a) in replicative senescence of normal human fibroblasts. *Proc. Natl. Acad. Sci. USA* 93, 13742-13747. 10.1073/pnas.93.24.13742 8943005 PMC19411

[BIO038257C2] Almeida A., Bolanos J. P. and Moncada S. (2010). E3 ubiquitin ligase APC/C-Cdh1 accounts for the Warburg effect by linking glycolysis to cell proliferation. *Proc. Natl. Acad. Sci. USA* 107, 738-741. 10.1073/pnas.0913668107 20080744 PMC2818939

[BIO038257C3] Bar-Peled L. and Sabatini D. M. (2014). Regulation of mTORC1 by amino acids. *Trends Cell Biol.* 24, 400-406. 10.1016/j.tcb.2014.03.003 24698685 PMC4074565

[BIO038257C4] Bauer D. E., Hatzivassiliou G., Zhao F., Andreadis C. and Thompson C. B. (2005). ATP citrate lyase is an important component of cell growth and transformation. *Oncogene* 24, 6314-6322. 10.1038/sj.onc.1208773 16007201

[BIO038257C5] Beausejour C. M., Krtolica A., Galimi F., Narita M., Lowe S. W., Yaswen P. and Campisi J. (2003). Reversal of human cellular senescence: roles of the p53 and p16 pathways. *EMBO J.* 22, 4212-4222. 10.1093/emboj/cdg417 12912919 PMC175806

[BIO038257C6] Beckman K. B. and Ames B. N. (1998). Mitochondrial aging: open questions. *Ann. N. Y. Acad. Sci.* 854, 118-127. 10.1111/j.1749-6632.1998.tb09897.x 9928425

[BIO038257C7] Ben-Porath I. and Weinberg R. A. (2005). The signals and pathways activating cellular senescence. *Int. J. Biochem. Cell Biol.* 37, 961-976. 10.1016/j.biocel.2004.10.013 15743671

[BIO038257C8] Borradaile N. M. and Pickering J. G. (2009). Nicotinamide phosphoribosyltransferase imparts human endothelial cells with extended replicative lifespan and enhanced angiogenic capacity in a high glucose environment. *Aging Cell* 8, 100-112. 10.1111/j.1474-9726.2009.00453.x 19302375

[BIO038257C9] Campisi J., Andersen J. K., Kapahi P. and Melov S. (2011). Cellular senescence: a link between cancer and age-related degenerative disease? *Semin. Cancer Biol.* 21, 354-359. 10.1016/j.semcancer.2011.09.001 21925603 PMC3230665

[BIO038257C10] Chang N., Yi J., Guo G., Liu X., Shang Y., Tong T., Cui Q., Zhan M., Gorospe M. and Wang W. (2010). HuR uses AUF1 as a cofactor to promote p16INK4 mRNA decay. *Mol. Cell. Biol.* 30, 3875-3886. 10.1128/MCB.00169-10 20498276 PMC2916395

[BIO038257C11] Chen Q. and Ames B. N. (1994). Senescence-like growth arrest induced by hydrogen peroxide in human diploid fibroblast F65 cells. *Proc. Natl. Acad. Sci. USA* 91, 4130-4134. 10.1073/pnas.91.10.4130 8183882 PMC43738

[BIO038257C12] Chen Q., Fischer A., Reagan J. D., Yan L. J. and Ames B. N. (1995). Oxidative DNA damage and senescence of human diploid fibroblast cells. *Proc. Natl. Acad. Sci. USA* 92, 4337-4341. 10.1073/pnas.92.10.4337 7753808 PMC41939

[BIO038257C13] Chen R., Zou Y., Mao D., Sun D., Gao G., Shi J., Liu X., Zhu C., Yang M., Ye W. et al. (2014). The general amino acid control pathway regulates mTOR and autophagy during serum/glutamine starvation. *J. Cell Biol.* 206, 173-182. 10.1083/jcb.201403009 25049270 PMC4107793

[BIO038257C14] Childs B. G., Baker D. J., Kirkland J. L., Campisi J. and van Deursen J. M. (2014). Senescence and apoptosis: dueling or complementary cell fates? *EMBO Rep.* 15, 1139-1153. 10.15252/embr.201439245 25312810 PMC4253488

[BIO038257C15] Cohen A. and Hall M. N. (2009). An amino acid shuffle activates mTORC1. *Cell* 136, 399-400. 10.1016/j.cell.2009.01.021 19203575

[BIO038257C16] de Magalhães J. P. and Passos J. F. (2018). Stress, cell senescence and organismal ageing. *Mech. Ageing Dev.* 170, 2-9. 10.1016/j.mad.2017.07.001 28688962

[BIO038257C17] DeBerardinis R. J., Mancuso A., Daikhin E., Nissim I., Yudkoff M., Wehrli S. and Thompson C. B. (2007). Beyond aerobic glycolysis: transformed cells can engage in glutamine metabolism that exceeds the requirement for protein and nucleotide synthesis. *Proc. Natl. Acad. Sci. USA* 104, 19345-19350. 10.1073/pnas.0709747104 18032601 PMC2148292

[BIO038257C18] DeBerardinis R. J., Lum J. J., Hatzivassiliou G. and Thompson C. B. (2008). The biology of cancer: metabolic reprogramming fuels cell growth and proliferation. *Cell Metab.* 7, 11-20. 10.1016/j.cmet.2007.10.002 18177721

[BIO038257C19] Dimri G. P. (2005). What has senescence got to do with cancer? *Cancer Cell* 7, 505-512. 10.1016/j.ccr.2005.05.025 15950900 PMC1769521

[BIO038257C20] Dimri G. P., Lee X., Basile G., Acosta M., Scott G., Roskelley C., Medrano E. E., Linskens M., Rubelj I., Pereira-Smith O. et al. (1995). A biomarker that identifies senescent human cells in culture and in aging skin in vivo. *Proc. Natl. Acad. Sci. USA* 92, 9363-9367. 10.1073/pnas.92.20.9363 7568133 PMC40985

[BIO038257C21] Duan S. and Pagano M. (2011). Linking metabolism and cell cycle progression via the APC/CCdh1 and SCFbetaTrCP ubiquitin ligases. *Proc. Natl. Acad. Sci. USA* 108, 20857-20858. 10.1073/pnas.1118443109 22173637 PMC3248501

[BIO038257C22] Estévez-García I. O., Cordoba-Gonzalez V., Lara-Padilla E., Fuentes-Toledo A., Falfán-Valencia R., Campos-Rodríguez R. and Abarca-Rojano E. (2014). Glucose and glutamine metabolism control by APC and SCF during the G1-to-S phase transition of the cell cycle. *J. Physiol. Biochem.* 70, 569-581. 10.1007/s13105-014-0328-1 24604252

[BIO038257C23] Fan J., Kamphorst J. J., Mathew R., Chung M. K., White E., Shlomi T. and Rabinowitz J. D. (2013). Glutamine-driven oxidative phosphorylation is a major ATP source in transformed mammalian cells in both normoxia and hypoxia. *Mol. Syst. Biol.* 9, 712 10.1038/msb.2013.65 24301801 PMC3882799

[BIO038257C24] Fingar D. C. and Blenis J. (2004). Target of rapamycin (TOR): an integrator of nutrient and growth factor signals and coordinator of cell growth and cell cycle progression. *Oncogene* 23, 3151-3171. 10.1038/sj.onc.1207542 15094765

[BIO038257C25] Gillies R. J., Didier N. and Denton M. (1986). Determination of cell number in monolayer cultures. *Anal. Biochem.* 159, 109-113. 10.1016/0003-2697(86)90314-3 3812988

[BIO038257C26] Goto M., Holgersson J., Kumagai-Braesch M. and Korsgren O. (2006). The ADP/ATP ratio: A novel predictive assay for quality assessment of isolated pancreatic islets. *Am. J. Transplant.* 6, 2483-2487. 10.1111/j.1600-6143.2006.01474.x 16869808

[BIO038257C27] Guertin D. A. and Sabatini D. M. (2007). Defining the role of mTOR in cancer. *Cancer Cell* 12, 9-22. 10.1016/j.ccr.2007.05.008 17613433

[BIO038257C28] Gwinn D. M., Shackelford D. B., Egan D. F., Mihaylova M. M., Mery A., Vasquez D. S., Turk B. E. and Shaw R. J. (2008). AMPK phosphorylation of raptor mediates a metabolic checkpoint. *Mol. Cell* 30, 214-226. 10.1016/j.molcel.2008.03.003 18439900 PMC2674027

[BIO038257C29] Hagiwara A., Cornu M., Cybulski N., Polak P., Betz C., Trapani F., Terracciano L., Heim M. H., Rüegg M. A. and Hall M. N. (2012). Hepatic mTORC2 activates glycolysis and lipogenesis through Akt, glucokinase, and SREBP1c. *Cell Metab.* 15, 725-738. 10.1016/j.cmet.2012.03.015 22521878

[BIO038257C30] Hashizume O., Ohnishi S., Mito T., Shimizu A., Iashikawa K., Nakada K., Soda M., Mano H., Togayachi S., Miyoshi H. et al. (2015). Epigenetic regulation of the nuclear-coded GCAT and SHMT2 genes confers human age-associated mitochondrial respiration defects. *Sci. Rep.* 5, 10434 10.1038/srep10434 26000717 PMC5377050

[BIO038257C31] Hatzivassiliou G., Zhao F., Bauer D. E., Andreadis C., Shaw A. N., Dhanak D., Hingorani S. R., Tuveson D. A. and Thompson C. B. (2005). ATP citrate lyase inhibition can suppress tumor cell growth. *Cancer Cell* 8, 311-321. 10.1016/j.ccr.2005.09.008 16226706

[BIO038257C32] Hayflick L. (1965). The limited in vitro lifetime of human diploid cell strains. *Exp. Cell Res.* 37, 614-636. 10.1016/0014-4827(65)90211-9 14315085

[BIO038257C33] Hayflick L. and Moorhead P. S. (1961). The serial cultivation of human diploid cell strains. *Exp. Cell Res.* 25, 585-621. 10.1016/0014-4827(61)90192-6 13905658

[BIO038257C34] Helmbold H., Komm N., Deppert W. and Bohn W. (2009). Rb2/p130 is the dominating pocket protein in the p53-p21 DNA damage response pathway leading to senescence. *Oncogene* 28, 3456-3467. 10.1038/onc.2009.222 19648966

[BIO038257C35] Hensley C. T., Wasti A. T. and DeBerardinis R. J. (2013). Glutamine and cancer: cell biology, physiology, and clinical opportunities. *J. Clin. Invest.* 123, 3678-3684. 10.1172/JCI69600 23999442 PMC3754270

[BIO038257C36] Ho C., van der Veer E., Akawi O. and Pickering J. G. (2009). SIRT1 markedly extends replicative lifespan if the NAD+ salvage pathway is enhanced. *FEBS Lett.* 583, 3081-3085. 10.1016/j.febslet.2009.08.031 19716821

[BIO038257C37] Hou P., Kuo C.-Y., Cheng C.-T., Liou J.-P., Ann D. K. and Chen Q. (2014). Intermediary metabolite precursor dimethyl-2-ketoglutarate stabilizes hypoxia-inducible factor-1alpha by inhibiting prolyl-4-hydroxylase PHD2. *PLoS ONE* 9, e113865 10.1371/journal.pone.0113865 25420025 PMC4242664

[BIO038257C38] Inoki K., Zhu T. and Guan K.-L. (2003). TSC2 mediates cellular energy response to control cell growth and survival. *Cell* 115, 577-590. 10.1016/S0092-8674(03)00929-2 14651849

[BIO038257C39] Jacinto E., Loewith R., Schmidt A., Lin S., Rüegg M. A., Hall A. and Hall M. N. (2004). Mammalian TOR complex 2 controls the actin cytoskeleton and is rapamycin insensitive. *Nat. Cell Biol.* 6, 1122-1128. 10.1038/ncb1183 15467718

[BIO038257C40] Jacinto E., Facchinetti V., Liu D., Soto N., Wei S., Jung S. Y., Huang Q., Qin J. and Su B. (2006). SIN1/MIP1 maintains rictor-mTOR complex integrity and regulates Akt phosphorylation and substrate specificity. *Cell* 127, 125-137. 10.1016/j.cell.2006.08.033 16962653

[BIO038257C41] Jiang P., Du W., Mancuso A., Wellen K. E. and Yang X. (2013). Reciprocal regulation of p53 and malic enzymes modulates metabolism and senescence. *Nature* 493, 689-693. 10.1038/nature11776 23334421 PMC3561500

[BIO038257C42] Jones R. G., Plas D. R., Kubek S., Buzzai M., Mu J., Xu Y., Birnbaum M. J. and Thompson C. B. (2005). AMP-activated protein kinase induces a p53-dependent metabolic checkpoint. *Mol. Cell* 18, 283-293. 10.1016/j.molcel.2005.03.027 15866171

[BIO038257C43] Julien L. A., Carriere A., Moreau J. and Roux P. P. (2010). mTORC1-activated S6K1 phosphorylates Rictor on threonine 1135 and regulates mTORC2 signaling. *Mol. Cell. Biol.* 30, 908-921. 10.1128/MCB.00601-09 19995915 PMC2815569

[BIO038257C44] Kaadige M. R., Looper R. E., Kamalanaadhan S. and Ayer D. E. (2009). Glutamine-dependent anapleurosis dictates glucose uptake and cell growth by regulating MondoA transcriptional activity. *Proc. Natl. Acad. Sci. USA* 106, 14878-14883. 10.1073/pnas.0901221106 19706488 PMC2736411

[BIO038257C45] Kalucka J., Missiaen R., Georgiadou M., Schoors S., Lange C., De Bock K., Dewerchin M. and Carmeliet P. (2015). Metabolic control of the cell cycle. *Cell Cycle* 14, 3379-3388. 10.1080/15384101.2015.1090068 26431254 PMC4825590

[BIO038257C46] Kaplon J., Zheng L., Meissl K., Chaneton B., Selivanov V. A., Mackay G., van der Burg S. H., Verdegaal E. M., Cascante M., Shlomi T. et al. (2013). A key role for mitochondrial gatekeeper pyruvate dehydrogenase in oncogene-induced senescence. *Nature* 498, 109-112. 10.1038/nature12154 23685455

[BIO038257C47] Kekuda R., Wang H., Huang W., Pajor A. M., Leibach F. H., Devoe L. D., Prasad P. D. and Ganapathy V. (1999). Primary structure and functional characteristics of a mammalian sodium-coupled high affinity dicarboxylate transporter. *J. Biol. Chem.* 274, 3422-3429. 10.1074/jbc.274.6.3422 9920886

[BIO038257C48] Kim S. G., Hoffman G. R., Poulogiannis G., Buel G. R., Jang Y. J., Lee K. W., Kim B.-Y., Erikson R. L., Cantley L. C., Choo A. Y. et al. (2013). Metabolic stress controls mTORC1 lysosomal localization and dimerization by regulating the TTT-RUVBL1/2 complex. *Mol. Cell* 49, 172-185. 10.1016/j.molcel.2012.10.003 23142078 PMC3545014

[BIO038257C49] Kujoth G. C., Hiona A., Pugh T. D., Someya S., Panzer K., Wohlgemuth S. E., Hofer T., Seo A. Y., Sullivan R., Jobling W. A. et al. (2005). Mitochondrial DNA mutations, oxidative stress, and apoptosis in mammalian aging. *Science* 309, 481-484. 10.1126/science.1112125 16020738

[BIO038257C50] Kumar A., Lawrence J. C. Jr, Jung D. Y., Ko H. J., Keller S. R., Kim J. K., Magnuson M. A. and Harris T. E. (2010). Fat cell-specific ablation of rictor in mice impairs insulin-regulated fat cell and whole-body glucose and lipid metabolism. *Diabetes* 59, 1397-1406. 10.2337/db09-1061 20332342 PMC2874700

[BIO038257C51] Langley E., Pearson M., Faretta M., Bauer U. M., Frye R. A., Minucci S., Pelicci P. G. and Kouzarides T. (2002). Human SIR2 deacetylates p53 and antagonizes PML/p53-induced cellular senescence. *EMBO J.* 21, 2383-2396. 10.1093/emboj/21.10.2383 12006491 PMC126010

[BIO038257C52] Laplante M. and Sabatini D. M. (2012). mTOR signaling in growth control and disease. *Cell* 149, 274-293. 10.1016/j.cell.2012.03.017 22500797 PMC3331679

[BIO038257C53] Lawless C., Jurk D., Gillespie C. S., Shanley D., Saretzki G., von Zglinicki T. and Passos J. F. (2012). A stochastic step model of replicative senescence explains ROS production rate in ageing cell populations. *PLoS ONE* 7, e32117 10.1371/journal.pone.0032117 22359661 PMC3281103

[BIO038257C54] Lee S. M., Dho S. H., Ju S.-K., Maeng J.-S., Kim J.-Y. and Kwon K.-S. (2012). Cytosolic malate dehydrogenase regulates senescence in human fibroblasts. *Biogerontology* 13, 525-536. 10.1007/s10522-012-9397-0 22971926

[BIO038257C55] Li J., Kim S. G. and Blenis J. (2014). Rapamycin: one drug, many effects. *Cell Metab.* 19, 373-379. 10.1016/j.cmet.2014.01.001 24508508 PMC3972801

[BIO038257C56] Liu W., Hong Q., Bai X.-Y., Fu B., Xie Y., Zhang X., Li J., Shi S., Lv Y., Sun X. et al. (2010). High-affinity Na(+)-dependent dicarboxylate cotransporter promotes cellular senescence by inhibiting SIRT1. *Mech. Ageing Dev.* 131, 601-613. 10.1016/j.mad.2010.08.006 20813124 PMC7127227

[BIO038257C57] Liu P., Guo J., Gan W. and Wei W. (2014). Dual phosphorylation of Sin1 at T86 and T398 negatively regulates mTORC2 complex integrity and activity. *Protein Cell* 5, 171-177. 10.1007/s13238-014-0021-8 24481632 PMC3967077

[BIO038257C58] Lunt S. Y. and Vander Heiden M. G. (2011). Aerobic glycolysis: meeting the metabolic requirements of cell proliferation. *Annu. Rev. Cell Dev. Biol.* 27, 441-464. 10.1146/annurev-cellbio-092910-154237 21985671

[BIO038257C59] Miyauchi H., Minamino T., Tateno K., Kunieda T., Toko H. and Komuro I. (2004). Akt negatively regulates the in vitro lifespan of human endothelial cells via a p53/p21-dependent pathway. *EMBO J.* 23, 212-220. 10.1038/sj.emboj.7600045 14713953 PMC1271675

[BIO038257C60] Mohrin M. and Chen D. (2016). The mitochondrial metabolic checkpoint and aging of hematopoietic stem cells. *Curr. Opin Hematol.* 23, 318-324. 10.1097/MOH.0000000000000244 26945277 PMC4891268

[BIO038257C61] Moiseeva O., Bourdeau V., Roux A., Deschenes-Simard X. and Ferbeyre G. (2009). Mitochondrial dysfunction contributes to oncogene-induced senescence. *Mol. Cell. Biol.* 29, 4495-4507. 10.1128/MCB.01868-08 19528227 PMC2725737

[BIO038257C62] Narita M., Nuñez S., Heard E., Narita M., Lin A. W., Hearn S. A., Spector D. L., Hannon G. J. and Lowe S. W. (2003). Rb-mediated heterochromatin formation and silencing of E2F target genes during cellular senescence. *Cell* 113, 703-716. 10.1016/S0092-8674(03)00401-X 12809602

[BIO038257C63] Nogueira V., Park Y., Chen C.-C., Xu P.-Z., Chen M.-L., Tonic I., Unterman T. and Hay N. (2008). Akt determines replicative senescence and oxidative or oncogenic premature senescence and sensitizes cells to oxidative apoptosis. *Cancer Cell* 14, 458-470. 10.1016/j.ccr.2008.11.003 19061837 PMC3038665

[BIO038257C64] Oh W. J. and Jacinto E. (2011). mTOR complex 2 signaling and functions. *Cell Cycle* 10, 2305-2316. 10.4161/cc.10.14.16586 21670596 PMC3322468

[BIO038257C65] Owen O. E., Kalhan S. C. and Hanson R. W. (2002). The key role of anaplerosis and cataplerosis for citric acid cycle function. *J. Biol. Chem.* 277, 30409-30412. 10.1074/jbc.R200006200 12087111

[BIO038257C66] Pajor A. M. (2014). Sodium-coupled dicarboxylate and citrate transporters from the SLC13 family. *Pflugers Arch.* 466, 119-130. 10.1007/s00424-013-1369-y 24114175

[BIO038257C67] Pizer E. S., Wood F. D., Heine H. S., Romantsev F. E., Pasternack G. R. and Kuhajda F. P. (1996). Inhibition of fatty acid synthesis delays disease progression in a xenograft model of ovarian cancer. *Cancer Res.* 56, 1189-1193. 8640795

[BIO038257C68] Qian Y. and Chen X. (2013). Senescence regulation by the p53 protein family. *Methods Mol. Biol.* 965, 37-61. 10.1007/978-1-62703-239-1_3 23296650 PMC3784259

[BIO038257C69] Sabatini D. M. (2006). mTOR and cancer: insights into a complex relationship. *Nat. Rev. Cancer* 6, 729-734. 10.1038/nrc1974 16915295

[BIO038257C70] Saci A., Cantley L. C. and Carpenter C. L. (2011). Rac1 regulates the activity of mTORC1 and mTORC2 and controls cellular size. *Mol. Cell* 42, 50-61. 10.1016/j.molcel.2011.03.017 21474067 PMC3750737

[BIO038257C71] Sahin E. and DePinho R. A. (2012). Axis of ageing: telomeres, p53 and mitochondria. *Nat. Rev. Mol. Cell Biol.* 13, 397-404. 10.1038/nrm3352 22588366 PMC3718675

[BIO038257C72] Sancak Y. and Sabatini D. M. (2009). Rag proteins regulate amino-acid-induced mTORC1 signalling. *Biochem. Soc. Trans.* 37, 289-290. 10.1042/BST0370289 19143648 PMC3390249

[BIO038257C73] Sarbassov D. D., Ali S. M., Kim D.-H., Guertin D. A., Latek R. R., Erdjument-Bromage H., Tempst P. and Sabatini D. M. (2004). Rictor, a novel binding partner of mTOR, defines a rapamycin-insensitive and raptor-independent pathway that regulates the cytoskeleton. *Curr. Biol.* 14, 1296-1302. 10.1016/j.cub.2004.06.054 15268862

[BIO038257C74] Sarbassov D. D., Ali S. M. and Sabatini D. M. (2005a). Growing roles for the mTOR pathway. *Curr. Opin. Cell Biol.* 17, 596-603. 10.1016/j.ceb.2005.09.009 16226444

[BIO038257C75] Sarbassov D. D., Guertin D. A., Ali S. M. and Sabatini D. M. (2005b). Phosphorylation and regulation of Akt/PKB by the rictor-mTOR complex. *Science* 307, 1098-1101. 10.1126/science.1106148 15718470

[BIO038257C76] Sarbassov D. D., Ali S. M., Sengupta S., Sheen J.-H., Hsu P. P., Bagley A. F., Markhard A. L. and Sabatini D. M. (2006). Prolonged rapamycin treatment inhibits mTORC2 assembly and Akt/PKB. *Mol. Cell* 22, 159-168. 10.1016/j.molcel.2006.03.029 16603397

[BIO038257C77] Shigenaga M. K., Hagen T. M. and Ames B. N. (1994). Oxidative damage and mitochondrial decay in aging. *Proc. Natl. Acad. Sci. USA* 91, 10771-10778. 10.1073/pnas.91.23.10771 7971961 PMC45108

[BIO038257C78] Shiota C., Woo J.-T., Lindner J., Shelton K. D. and Magnuson M. A. (2006). Multiallelic disruption of the rictor gene in mice reveals that mTOR complex 2 is essential for fetal growth and viability. *Dev. Cell* 11, 583-589. 10.1016/j.devcel.2006.08.013 16962829

[BIO038257C79] Sohal R. S. and Weindruch R. (1996). Oxidative stress, caloric restriction, and aging. *Science* 273, 59-63. 10.1126/science.273.5271.59 8658196 PMC2987625

[BIO038257C80] Stein G. H., Drullinger L. F., Soulard A. and Dulić V. (1999). Differential roles for cyclin-dependent kinase inhibitors p21 and p16 in the mechanisms of senescence and differentiation in human fibroblasts. *Mol. Cell. Biol.* 19, 2109-2117. 10.1128/MCB.19.3.2109 10022898 PMC84004

[BIO038257C81] Stöckl P., Hütter E., Zwerschke W. and Jansen-Dürr P. (2006). Sustained inhibition of oxidative phosphorylation impairs cell proliferation and induces premature senescence in human fibroblasts. *Exp. Gerontol.* 41, 674-682. 10.1016/j.exger.2006.04.009 16713693

[BIO038257C82] Straface E., Vona R., Ascione B., Matarrese P., Strudthoff T., Franconi F. and Malorni W. (2007). Single exposure of human fibroblasts (WI-38) to a sub-cytotoxic dose of UVB induces premature senescence. *FEBS Lett.* 581, 4342-4348. 10.1016/j.febslet.2007.08.006 17716665

[BIO038257C83] Strohecker A. M. and White E. (2014). Autophagy promotes BrafV600E-driven lung tumorigenesis by preserving mitochondrial metabolism. *Autophagy* 10, 384-385. 10.4161/auto.27320 24362353 PMC5396093

[BIO038257C84] Takahashi A., Ohtani N., Yamakoshi K., Iida S., Tahara H., Nakayama K., Nakayama K. I., Ide T., Saya H. and Hara E. (2006). Mitogenic signalling and the p16INK4a-Rb pathway cooperate to enforce irreversible cellular senescence. *Nat. Cell Biol.* 8, 1291-1297. 10.1038/ncb1491 17028578

[BIO038257C85] Taylor J. R., Lehmann B. D., Chappell W. H., Abrams S. L., Steelman L. S. and McCubrey J. A. (2011). Cooperative effects of Akt-1 and Raf-1 on the induction of cellular senescence in doxorubicin or tamoxifen treated breast cancer cells. *Oncotarget* 2, 610-626. 10.18632/oncotarget.315 21881167 PMC3248208

[BIO038257C86] Thoreen C. C., Kang S. A., Chang J. W., Liu Q., Zhang J., Gao Y., Reichling L. J., Sim T., Sabatini D. M. and Gray N. S. (2009). An ATP-competitive mammalian target of rapamycin inhibitor reveals rapamycin-resistant functions of mTORC1. *J. Biol. Chem.* 284, 8023-8032. 10.1074/jbc.M900301200 19150980 PMC2658096

[BIO038257C87] Trifunovic A., Hansson A., Wredenberg A., Rovio A. T., Dufour E., Khvorostov I., Spelbrink J. N., Wibom R., Jacobs H. T. and Larsson N. G. (2005). Somatic mtDNA mutations cause aging phenotypes without affecting reactive oxygen species production. *Proc. Natl. Acad. Sci. USA* 102, 17993-17998. 10.1073/pnas.0508886102 16332961 PMC1312403

[BIO038257C88] van der Veer E., Ho C., O'Neil C., Barbosa N., Scott R., Cregan S. P. and Pickering J. G. (2007). Extension of human cell lifespan by nicotinamide phosphoribosyltransferase. *J. Biol. Chem.* 282, 10841-10845. 10.1074/jbc.C700018200 17307730

[BIO038257C89] Velarde M. C., Flynn J. M., Day N. U., Melov S. and Campisi J. (2012). Mitochondrial oxidative stress caused by Sod2 deficiency promotes cellular senescence and aging phenotypes in the skin. *Aging (Albany NY)* 4, 3-12. 10.18632/aging.100423 22278880 PMC3292901

[BIO038257C90] von Zglinicki T., Saretzki G., Döcke W. and Lotze C. (1995). Mild hyperoxia shortens telomeres and inhibits proliferation of fibroblasts: a model for senescence? *Exp. Cell Res.* 220, 186-193. 10.1006/excr.1995.1305 7664835

[BIO038257C91] Wallace D. C. (1999). Mitochondrial diseases in man and mouse. *Science* 283, 1482-1488. 10.1126/science.283.5407.1482 10066162

[BIO038257C92] Wang W., Yang X., López de Silanes I., Carling D. and Gorospe M. (2003). Increased AMP:ATP ratio and AMP-activated protein kinase activity during cellular senescence linked to reduced HuR function. *J. Biol. Chem.* 278, 27016-27023. 10.1074/jbc.M300318200 12730239

[BIO038257C93] Wiley C. D., Velarde M. C., Lecot P., Liu S., Sarnoski E. A., Freund A., Shirakawa K., Lim H. W., Davis S. S., Ramanathan A. et al. (2016). Mitochondrial Dysfunction Induces Senescence with a Distinct Secretory Phenotype. *Cell Metab.* 23, 303-314. 10.1016/j.cmet.2015.11.011 26686024 PMC4749409

[BIO038257C94] Wise D. R. and Thompson C. B. (2010). Glutamine addiction: a new therapeutic target in cancer. *Trends Biochem. Sci.* 35, 427-433. 10.1016/j.tibs.2010.05.003 20570523 PMC2917518

[BIO038257C95] Wise D. R., DeBerardinis R. J., Mancuso A., Sayed N., Zhang X.-Y., Pfeiffer H. K., Nissim I., Daikhin E., Yudkoff M., McMahon S. B. et al. (2008). Myc regulates a transcriptional program that stimulates mitochondrial glutaminolysis and leads to glutamine addiction. *Proc. Natl. Acad. Sci. USA* 105, 18782-18787. 10.1073/pnas.0810199105 19033189 PMC2596212

[BIO038257C96] Wu D. and Yotnda P. (2011). Production and detection of reactive oxygen species (ROS) in cancers. *J. Vis. Exp.* 57, e3357 10.3791/3357 PMC3308605 22127014

[BIO038257C97] Yao Y., Jones E. and Inoki K. (2017). Lysosomal regulation of mTORC1 by amino acids in mammalian cells. *Biomolecules* 7, E51 10.3390/biom7030051 28686218 PMC5618232

[BIO038257C98] Ye J., Palm W., Peng M., King B., Lindsten T., Li M. O., Koumenis C. and Thompson C. B. (2015). GCN2 sustains mTORC1 suppression upon amino acid deprivation by inducing Sestrin2. *Genes Dev.* 29, 2331-2336. 10.1101/gad.269324.115 26543160 PMC4691887

[BIO038257C99] Yuneva M., Zamboni N., Oefner P., Sachidanandam R. and Lazebnik Y. (2007). Deficiency in glutamine but not glucose induces MYC-dependent apoptosis in human cells. *J. Cell Biol.* 178, 93-105. 10.1083/jcb.200703099 17606868 PMC2064426

[BIO038257C100] Zhang R., Chen W. and Adams P. D. (2007). Molecular dissection of formation of senescence-associated heterochromatin foci. *Mol. Cell. Biol.* 27, 2343-2358. 10.1128/MCB.02019-06 17242207 PMC1820509

[BIO038257C101] Zoncu R., Efeyan A. and Sabatini D. M. (2011). mTOR: from growth signal integration to cancer, diabetes and ageing. *Nat. Rev. Mol. Cell Biol.* 12, 21-35. 10.1038/nrm3025 21157483 PMC3390257

