# Supplementary material for: Blockage of glutamine-dependent anaplerosis affects mTORC1/2 activity and ultimately leads to cellular senescence-like response
Source: Biol Open. 2019 May 16;8(5):bio038257. doi: 10.1242/bio.038257 (PMC6550068; doi:10.1242/bio.038257)
Supplement: Supplementary information [file biolopen-8-038257-s1.pdf]

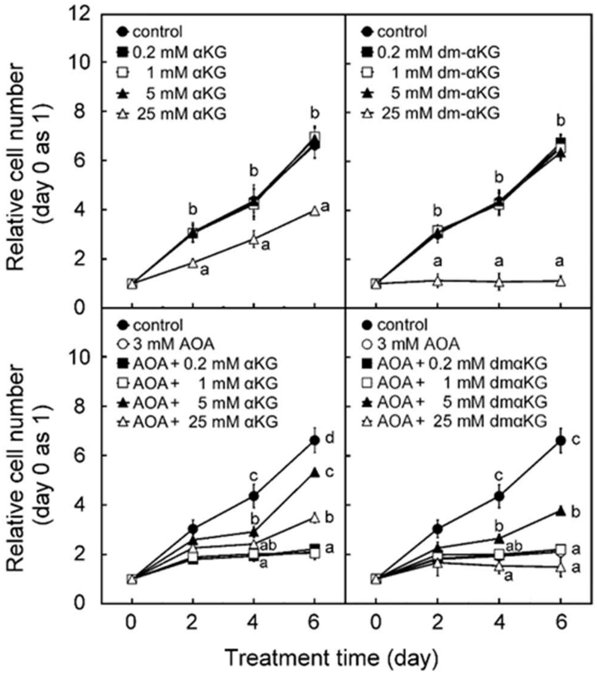

Figure S1. Effect of α-ketoglutarate (αKG) and analog dimethyl α-ketoglutarate (dmαKG) on AOA-induced inhibition of proliferation in WI38 cells. Cells were treated with vehicle or 3 mM AOA in the absence or presence of different concentrations (0.2, 1, 5, 25 mM) of dmαKG or αKG for the indicated time period with medium changed at 2-day intervals. Cell numbers were measured by crystal violet staining assay. Data are expressed as the mean±s.e.m. (n=3) of three independent experiments. Different lowercase letters indicate significant difference among treatment groups at the same time point ($P<0.05$).

Biology Open • Supplementary information

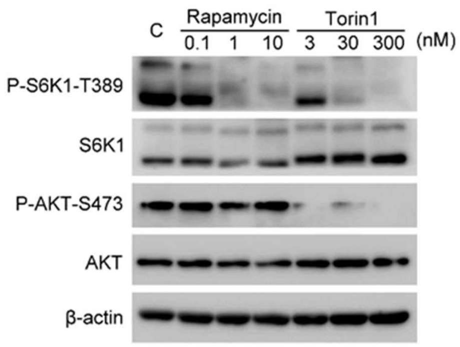

Figure S2. Dose-responsive effect of mTOR inhibitors (rapamycin and Torin1) on mTORC1/2 activity in WI38 cells. Cells were treated for 1 hour with control vehicle (DMSO), or different concentrations of rapamycin (0.1, 1, or 10 nM) or Torin1 (3, 30, or 300 nM). Cell lysates were prepared and analyzed by immunoblotting for P-S6K1-T389, S6K1, and P-AKT-S473 and AKT with β-actin served as a loading control.

Biology Open • Supplementary information

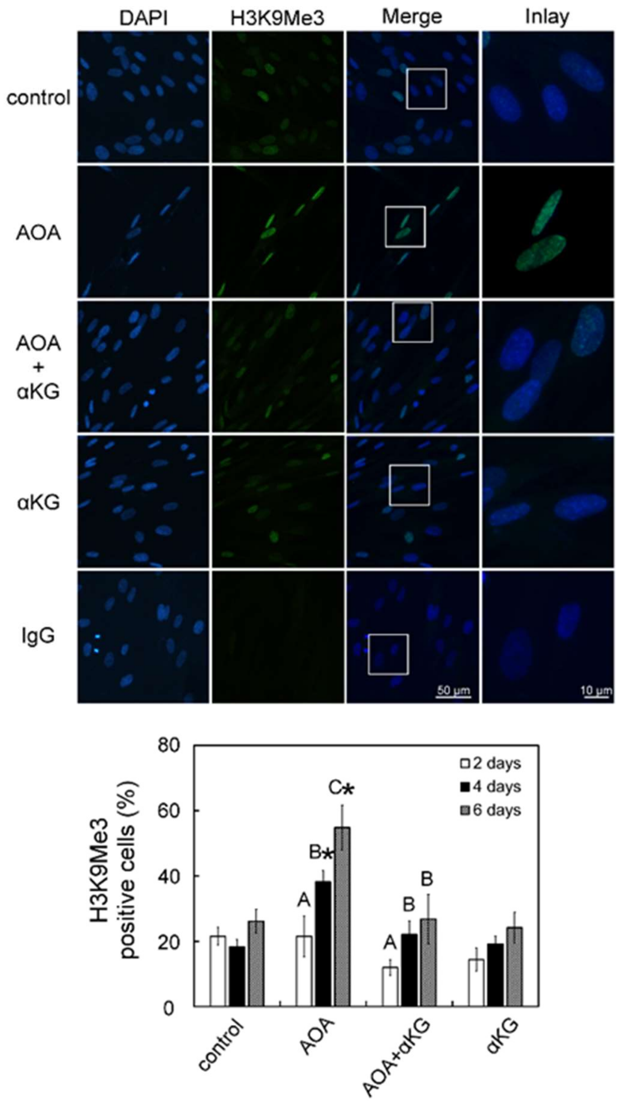

Figure S3. Prolonged blockade of glutamine-dependent anaplerosis with AOA induced senescence-associated marker H3K9Me3 in WI38 cells. Cells were treated with vehicle or AOA in the absence or presence of αKG for the indicated time period with medium changed at 2-day intervals. At the end of culture, cells were prepared for immunofluorescence staining of H3K9Me3 (green) and co-stained for nucleus with DAPI. (A) Representative microscopic cell images of all groups and negative IgG control, and (B) quantitation of the percentage of H3K9Me3-positive cells. Each sample was counted for 200 cells. Data are expressed as the mean±s.e.m. (n=3) of three independent experiments. Different uppercase letters indicate significant difference of the same treatment group at different time points ($P<0.05$). Asterisk (*) designates a significant difference compared with the respective vehicle control at the same time point ($P<0.05$).

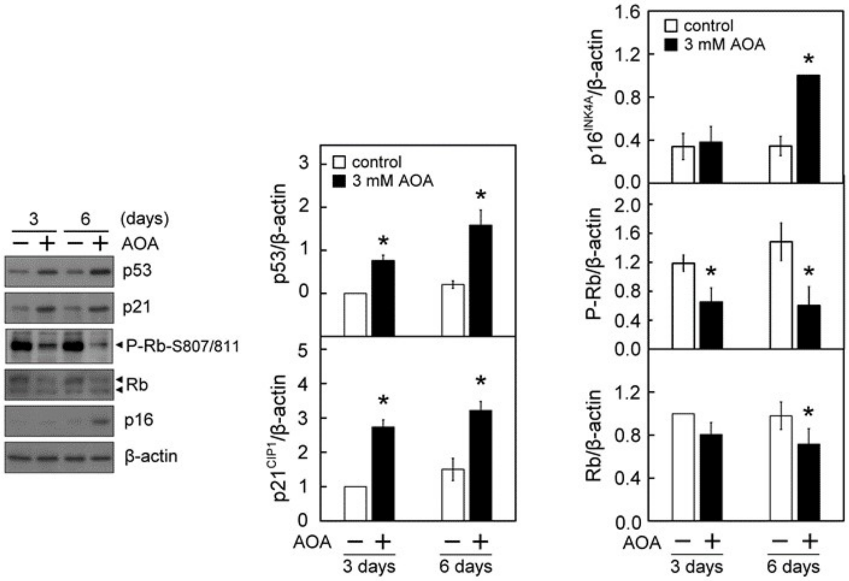

Figure S4. Temporal effect of AOA on senescence-inducing regulators in WI38 cells. Cells were treated 3 mM AOA for 3 and 6 days, and then cell lysates were prepared and analyzed by immunoblotting and densitometry analysis for p53, p21^{CIP1}, Rb, P-Rb-S807/811 and p16^{INK4A} with β-actin served as a loading control. All quantitative data are expressed as the mean±s.e.m. (n=3) of three independent experiments. Asterisk (*) designates a significant difference compared with the respective vehicle control (P<0.05).

Biology Open • Supplementary information

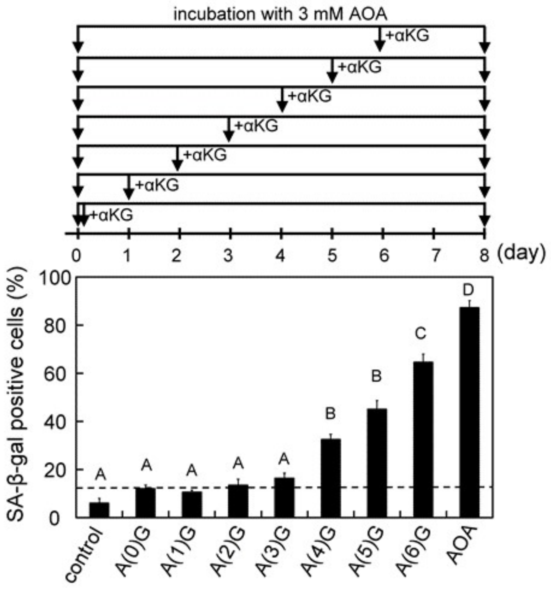

Figure S5. Effect of delayed αKG supplementation on the AOA-induced SA-β-gal activity in WI38 cells. Cell were treated with 3 mM AOA, and additional given with or without 5 mM αKG at the indicated time point post-AOA treatment. At the end of 8-day culture, the cells were analyzed for SA-β-gal activity as described in Methods. A(0-6)G indicates the time point when cells were given αKG. All quantitative data are expressed as the mean±s.e.m. (n=3) of three independent experiments. Different lowercase letters indicate significant difference among treatment groups ($P<0.05$).
